# Conditional knockout of Dkk3 drives Lgr5+ progenitor reprogramming into hair cells in the mouse cochlea

**DOI:** 10.7150/thno.131808

**Published:** 2026-05-18

**Authors:** Hairong Xiao, Xinlin Wang, Zixuan Ye, Xin Tan, Ying Ma, Xiangyu Ma, Wei Tong, Luying Zhang, Yanqin Lin, Xujun Tang, Huiling Zhang, Jinxian Wan, Qiuyue Zhang, Renjie Chai, Shasha Zhang

**Affiliations:** 1 State Key Laboratory of Digital Medical Engineering, Department of Otolaryngology Head and Neck Surgery, Zhongda Hospital, School of Life Sciences and Technology, Advanced Institute for Life and Health, Jiangsu Province High-Tech Key Laboratory for Bio-Medical Research, Southeast University, Nanjing, China.; 2 Southeast University Shenzhen Research Institute, Shenzhen, China.; 3 Department of Neurology, Aerospace Center Hospital, School of Life Science, Beijing Institute of Technology, Beijing, China.; 4 Co-innovation Center of Neuroregeneration, Nantong University, Nantong, China.; 5 Institute for Stem Cell and Regeneration, Chinese Academy of Science, Beijing, China.; 6 Beijing Key Laboratory of Neural Regeneration and Repair, Capital Medical University, Beijing, 100069, China.; 7 Department of Otolaryngology – Head and Neck Surgery, Stanford University School of Medicine, Stanford, CA 94305, USA.

**Keywords:** hair cell, Lgr5, cochlear plasticity, Dkk3, trans-differentiation

## Abstract

**Background:**

Neonatal cochlear Lgr5+ progenitors possess a transient regenerative capacity that diminishes rapidly after birth, severely limiting the potential for hearing restoration. Identifying the molecular mechanisms that restrict this plasticity is critical for developing effective regenerative therapies to treat hearing loss (HL).

**Methods:**

To uncover regulators of cochlear progenitor plasticity, we performed an integrative transcriptomic analysis across spatial, injury, and lineage contexts. Following the identification of candidate regulators, we investigated the function of *Dkk3*, a canonical Wnt antagonist, using *in vitro* Lgr5+ sphere formation assays and *in vivo* conditional knockout (cKO) models in neonatal progenitors. We assessed hair cell (HC) generation and maturation using lineage tracing, histological analysis of stereocilia and synapses, and electrophysiological recordings. Furthermore, we employed single-nucleus transcriptomics (snRNA-seq) of the *Dkk3* cKO cochlea to elucidate the underlying molecular signaling networks.

**Results:**

Our screen identified 14 candidate regulators, highlighting *Dkk3* as a previously uncharacterized factor in the auditory epithelium that functions as a physiological gatekeeper of Lgr5+ progenitor plasticity. *In vitro*, *Dkk3* knockdown significantly enhanced Lgr5+ sphere formation. *In vivo*, *Dkk3* cKO induced the spontaneous generation of HCs through direct trans-differentiation. Crucially, these ectopic HCs achieved structural maturity, characterized by organized stereocilia and synaptic connections, displayed partial electrophysiological activity, and survived long-term into adulthood without disrupting native auditory function. Mechanistically, snRNA-seq analysis and RT-qPCR validation suggested that *Dkk3* cKO may activate a pro-regenerative network involving the Wnt, Hedgehog, and mTOR signaling pathways.

**Conclusions:**

Our findings establish *Dkk3* as a key molecular inhibitor of sensory fate reprogramming in the cochlea. These results suggest that targeting *Dkk3* represents a promising therapeutic strategy for functional and durable HC reprogramming.

## Introduction

Sensorineural hearing loss (SNHL) affects millions worldwide, compromising quality of life and imposing substantial socioeconomic burdens^1^. However, no pharmacological therapies have been developed to treat or effectively reverse sensory deficits^2^. Cochlear hair cells (HCs) are mechanosensory receptors critical for auditory transduction, converting sound vibrations into neural signals that enable hearing^3^. One of the main causes of permanent HL is the degeneration of sensory HCs in the inner ear, which typically occurs due to aging, ototoxic agents, and noise-induced trauma^4^, ^5^. Non-mammalian vertebrates regenerate HCs throughout their lifespan via proliferation and differentiation of supporting cells (SC) following injury^6^, ^7^. Although mammal SCs retain some regenerative potential during early development and in response to injury in the neonatal cochlea, this capacity is almost lost in adult mammals, presenting a fundamental barrier to restoring hearing function following HC loss^8^-^10^.

The discovery of *Lgr5*+ cochlear progenitors with innate regenerative potential marked a turning point in hearing research^11^. These progenitors are capable of differentiating into HCs under specific conditions, a finding that has opened promising avenues for hearing restoration^12^-^14^. However, existing approaches have achieved only partial success, such as overexpression of pro-regenerative genes like* Atoh1*^15^ or modulation of signaling pathways like Wnt and Notch^16^. The HCs induced by these methods are frequently transient and structurally immature, often failing to establish synaptic connections or undergoing rapid apoptosis^17^-^19^. Recent evidence demonstrates that optimized strategies, such as the co-expression of* Atoh1*, *Gfi1*, and *Pou4f3*, or reprogramming via drug-like molecules, can successfully drive HC maturation and synaptic integration^19^-^21^. The need to deliver multiple genetic factors still strains the limits of clinical vector capacity. Consequently, identifying novel upstream regulators to optimize this process remains critical for achieving effective hearing restoration.

To systematically identify the molecular mechanisms restricting progenitor plasticity, we conducted an intersectional transcriptomic analysis integrating three complementary RNA-seq datasets. We compared: (1) apical *Lgr5*+ progenitors (ALPs) and basal *Lgr5*+ progenitors (BLPs) to probe spatial heterogeneity and the intrinsic regenerative competence of the apex; (2) untreated *Lgr5*+ progenitors (ULPs) and neomycin-treated *Lgr*5+ progenitors (NLPs) to capture the acute transcriptional response to damage; and (3) *Lgr5*+ progenitors vs. *Lgr5*- SC to define the core lineage identity^22^-^24^. This screening strategy identified 14 candidate regulators consistently associated with regenerative competence across all three contexts. Among these, we focused on *Dkk3*, a canonical Wnt antagonist whose function in the auditory epithelium remained unexplored.

Here, we identify *Dkk3* as a critical physiological negative regulator of cochlear Lgr5+ progenitor plasticity. We show that *Dkk3* knockdown enhances the *Lgr5*+ sphere-forming efficiency *in vitro*. In transgenic *Dkk3* conditional knockout (cKO) mice, we demonstrate that *Dkk3* cKO in *Lgr5*+ progenitors drives the spontaneous generation of HCs via direct trans-differentiation. Crucially, unlike the transient cells often reported in single-factor studies, the ectopic HCs generated in *Dkk3* cKO mice achieve molecular and structural differentiation with organized stereocilia and synaptic connections. Furthermore, these cells possess partial electrophysiological activity and survive long-term into adulthood without inducing deleterious effects on native auditory function. Single-nucleus RNA sequencing (snRNA-seq) of *Dkk3* cKO cochlear, supported by targeted validation, further revealed activation of a pro-regenerative network involving Wnt, Hedgehog, and mTOR signaling pathways. These findings establish *Dkk3* knockout as a robust strategy for inducing stable cellular reprogramming, providing a compelling rationale for future combinatorial treatments aimed at functional hearing restoration.

## Results

### Integrative transcriptomics identifies *Dkk3* as a negative regulator of cochlear progenitor plasticity

**Figure 1:**
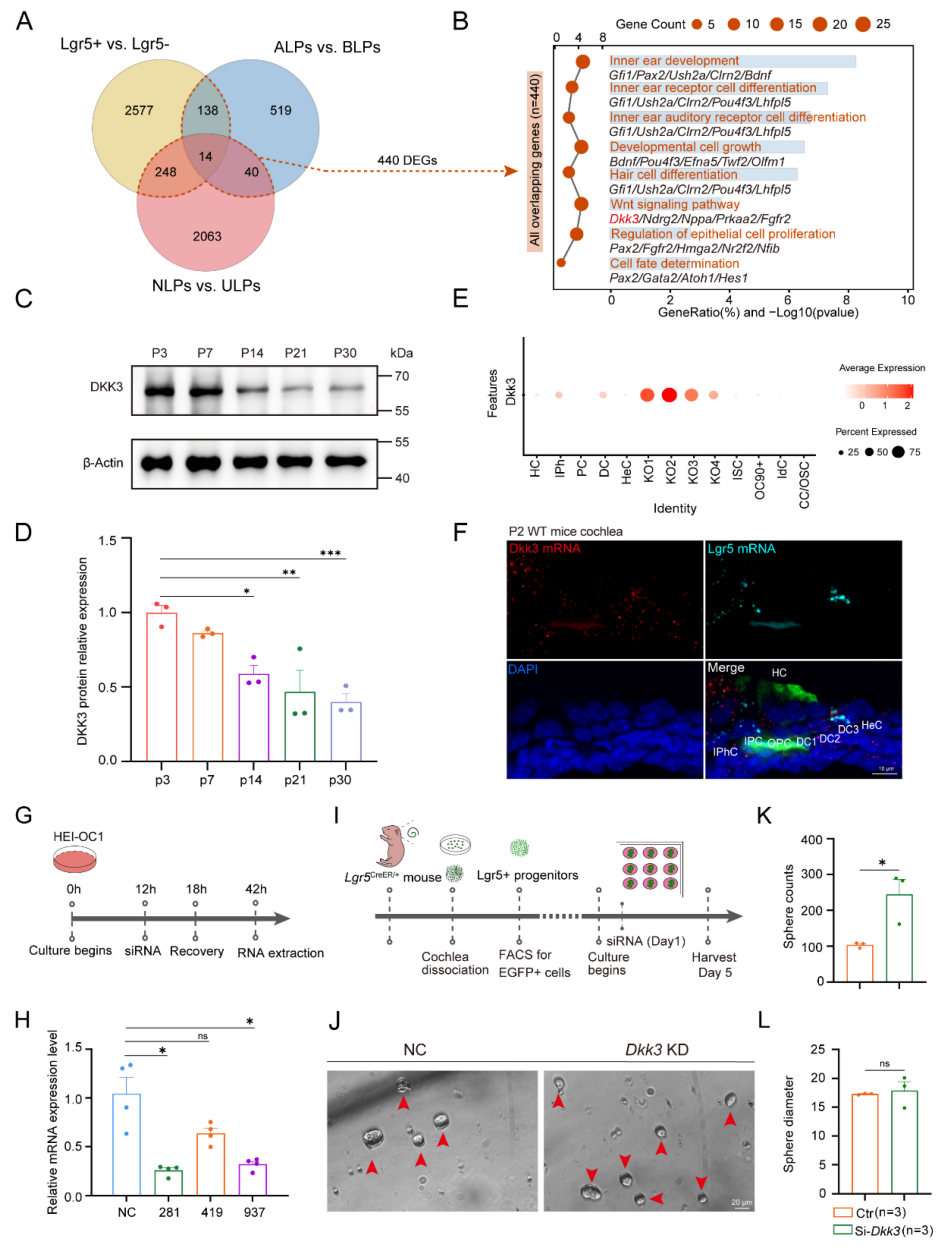
** Dkk3 expression pattern and sphere assay of Lgr5+ progenitors *in vitro* after *Dkk3* knockdown. (A)** Venn diagram of DEGs across three RNA-seq datasets. The dashed line indicates the 440 overlapping genes used for GO analysis, while the central intersection represents the 14 core candidates. **(B)** GO enrichment analysis of the 440 overlapping genes identified in (A). Representative genes for each pathway are listed. **(C-D)** Western blot (C) and quantification (D) of Dkk3 protein levels from P3 to P30 cochlea. **(E)** Dot plot showing *Dkk3* expression across cell clusters in P1 mouse cochlea. **(F)** Dual-probe RNAscope* in situ* hybridization of *Dkk3* (red) and *Lgr5* (cyan), combined with immunofluorescence for Myosin7a (green), in the OC of P2 WT cochlea; KO, Kölliker’s organ cells; IBC, Inner border cells; IPhC, inner phalangeal cells; IPC, Inner pillar cells; OPC, Outer pillar cells; DC, Deiters’ cells; IHC, Inner hair cells; OHC, Outer hair cells. **(G)** Workflow for siRNA-*Dkk3* knockdown validation in HEI-OC1 cells. **(H)** RT-qPCR validation of *Dkk3* knockdown (KD) efficiency using three siRNAs. **(I)** Schematic of Lgr5+ progenitor sphere assay. Lgr5+ progenitors were cultured *in vitro* for 5 days to form spheres after siRNA transfection. **(J)** Representative brightfield images of spheres following *Dkk3* KD and negative control (NC). Scale bar, 20 μm. **(K-L)** Quantification of sphere number (I) and diameter (J). * *p*<0.05, **p < 0.01, *** p < 0.001, and *ns*, not significant. “n” means biological replicates.

To identify core molecular mechanisms that restrict cochlear progenitor plasticity, we performed an intersectional transcriptomic analysis integrating three complementary RNA-seq datasets previously generated by our group. These datasets represented distinct axes of plasticity: spatial heterogeneity (ALPs vs. BLPs), injury response (NLPs vs. ULPs), and lineage identity (*Lgr5*+ Progenitors vs. *Lgr5*- SCs). Using uniform filtering thresholds (fold change > 2.0, *p* < 0.05), this cross-analysis narrowed down 440 overlapping genes to a core set of 14 candidates consistently differentially expressed across all three comparisons** (Figure 1A; Supplemental Table 1)**. To understand the biological landscape of these shared signatures, we performed Gene Ontology (GO) enrichment analysis on this 440-gene set. The results highlighted biological processes related to inner ear development, cell differentiation, and Wnt signaling pathways** (Figure 1B)**. From this broader set, we further narrowed our focus to a core set of 14 candidates that were consistently differentially expressed across all three comparisons. Notably, the 14 shared genes included known deafness-associated mutants (*Otoa*, *Col9a1*)^25^-^27^ and regulators of cell growth and differentiation (*Net1*, *Serpine2*, *Ndrg2*)^28^-^32^. Other candidate factors include transcription factors (*Fos*, *Fosb*)^33^, extracellular matrix and adhesion molecules (*Cntn1*, *Cyr61*)^34^, ^35^, and the calcium-binding protein *Capsl^36^*. Additionally, we identified key metabolic or transport regulators, specifically the metalloendopeptidase* Mme*, known for peptide metabolism^37^, and the GABA transporter *Slc6a11*, essential for mediating GABA reuptake and regulating tonic inhibition^38^.

Among these candidates, we prioritized *Dkk3*, a canonical Wnt antagonist whose physiological function in the auditory epithelium remained unexplored^39^. In wild-type mice, Dkk3 protein levels declined more than 2-fold from postnatal day (P) 3 to P30 **(Figure 1C, D)**, a temporal pattern coinciding with the progressive maturation of the organ of Corti and the loss of regenerative potential. snRNA-seq data^40^ and RNAscope *in situ* hybridization localized *Dkk3* to the Organ of Corti (OC), including inner phalangeal (IPhC), Deiters’ (DC), and pillar cells (PC) in P2 wild-type (WT) mice cochlea. Notably, RNAscope *in situ* hybridization demonstrated that *Dkk3* and *Lgr5* primarily co-localize in the third row of DCs **(Figure 1E, F; Figure S1)**. Consistent with this, re-analysis of human fetal cochlea at 15 and 17 weeks revealed conserved *Dkk3* expression in DC and outer pillar cells (OPC)^41^ **(Figure S2)**.

To investigate whether *Dkk3* functionally restricts progenitor plasticity, we utilized the *in vitro Lgr5*+ sphere-forming assay, an established surrogate for assessing stemness and regenerative capacity^42^. We first validated *Dkk3* knockdown efficiency in HEI-OC1 cells, identifying siRNA-281 as the most efficient knockdown construct in HEI-OC1 cells **(Figure 1G, H)**. Next, we FACS-isolated *Lgr5*-EGFP+ progenitors from P1 neonatal mice and cultured them under suspension conditions **(Figure 1I)**. *Dkk3* knockdown significantly enhanced the sphere-forming efficiency, increasing the number of spheres by more than two-fold without affecting organoid size **(Figure 1J-L)**. These findings nominate *Dkk3* as a key regulator of *Lgr5*+ progenitor plasticity and a promising candidate for modulating HC generation *in vivo*.

### *Dkk3* cKO in *Lgr5*+ progenitors triggers numerous ectopic HCs* in vivo*

To further explore the role of *Dkk3 in vivo*, *Dkk3*^flox^ mice were generated **(Figure S3A)** and validated successful recombination via genotyping **(Figure S3B)**. Tamoxifen was intraperitoneally (*i.p.*) administered at P1 to induce *Dkk3* knockout specifically in *Lgr5*+ progenitors **(Figure S3C)**. Both mRNA and protein levels of Dkk3 were significantly reduced in *Dkk3* cKO mice compared to controls **(Figure S3D-F)**, confirming efficient gene knockout. Furthermore, RNAscope* in situ* hybridization confirmed the specific depletion of Dkk3 mRNA signal within the Lgr5-positive cellular regions, particularly in the third row of DCs (**Figure S3G**).

**Figure 2:**
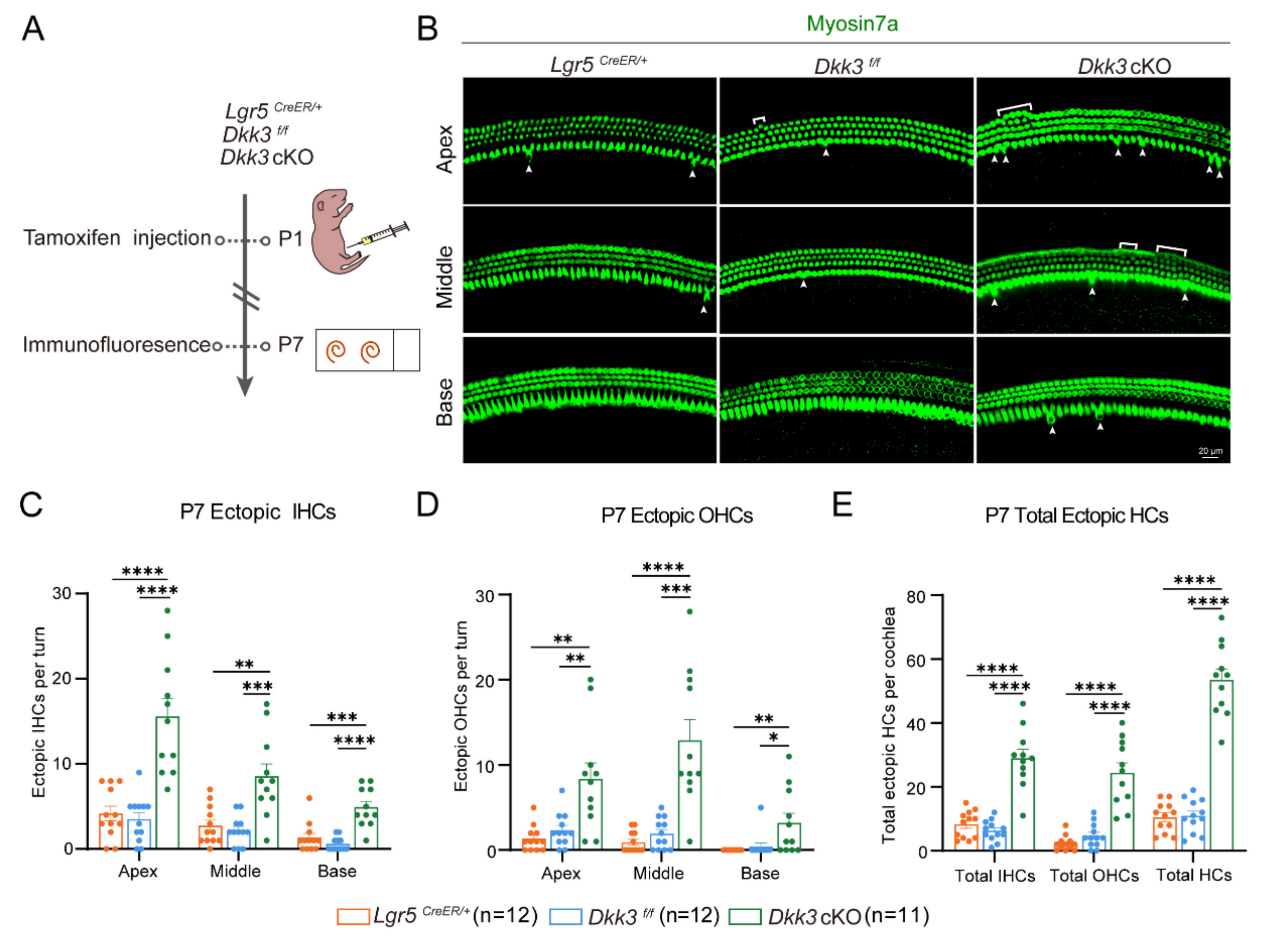
***Dkk3* cKO promotes ectopic HC regeneration in the neonatal mouse cochlea. (A)** Experimental workflow for Tamoxifen injection and cochlear imaging of *Dkk3* cKO mice. **(B)** Myosin7a-stained confocal images of P7 cochleae showing regenerated IHCs (arrows) and OHCs (brackets) in *Dkk3* cKO mice and control groups. Scale bars, 20 µm. **(C-E)** Quantitative analysis of ectopic IHCs per turn (C), ectopic OHCs per turn (D), and total ectopic HCs per cochlea (E). *p < 0.05, **p < 0.01, ***p < 0.001, ****p < 0.0001. “n” means biological replicates.

We next assessed the regenerative capacity of *Dkk3* cKO at P7 **(Figure 2A)**. Compared to* Lgr5*^CreER/+^ and *Dkk3*^f/f^ controls, *Dkk3* cKO mice exhibited numerous ectopic IHCs and OHCs in three turns of the cochlea (**Figure 2B**). *Dkk3* cKO mice exhibited a basal-to-apical increase gradient in ectopic IHC numbers across the cochlea, with fold increases of at least 8-fold (base), 3-fold (middle), and 4-fold (apex) relative to *Dkk3*^f/f^ control** (Figure 2C)**. The number of ectopic OHCs in three cochlear turns of *Dkk3* cKO mice was more than 4-fold, 6-fold, and 7-fold of that of *Dkk3*^f/f^ mice, respectively **(Figure 2D)**. These results demonstrate that *Dkk3* knockout in Lgr5+ progenitors is sufficient to trigger ectopic HC formation **(Figure 2E)**.

### *Dkk3* cKO drives hair cell spontaneous generation via direct trans-differentiation of *Lgr5*+ progenitors

HC regeneration generally occurs through two main mechanisms: (1) direct trans-differentiation of SCs and/or (2) mitotic regeneration involving proliferation and differentiation cascades^13^, ^43^. While our earlier* in vitro* work showed that *Dkk3* KD could boost *Lgr5*+ sphere-forming efficiency, the generative pathway *in vivo* remained unclear.

**Figure 3:**
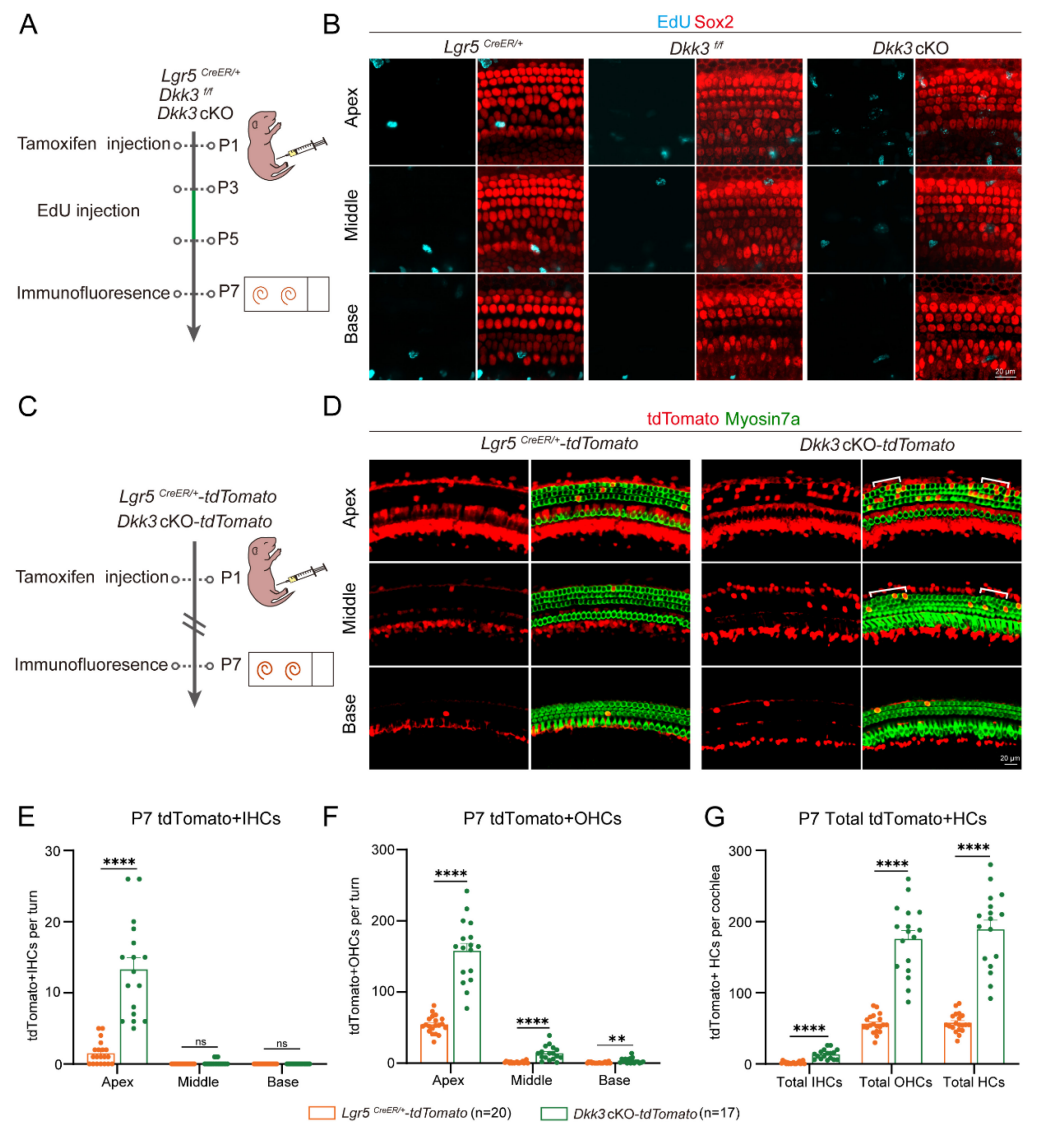
**EdU assay and lineage tracing of Lgr5+ progenitors in *Dkk3* cKO mice cochlea. (A)** Experimental workflow for EdU proliferation assay of *Dkk3* cKO mice. **(B)** Confocal images of EdU staining in *Dkk3* cKO mice and control groups' cochleae, immunolabeled with Sox2 to mark SCs. *Lgr5*^CreER/+^ mice and *Dkk3*^f/f^ mice were used as control groups. Scale bars, 20 µm. **(C)** Schematic for lineage tracing assay of *Dkk3* cKO mice.** (D)** Lineage tracing images of tdTomato+ HCs in the cochlea of *Dkk3* cKO-td*Tomato* mice cochleae and control groups.* Lgr5*^CreER/+^-td*Tomato* mice were used as the control group. Myosin7a was used as the HC marker. Scale bars, 20 µm. **(E-G)** Quantitative analysis of tdTomato+ IHCs in each turn (E), tdTomato+ OHCs in each turn (F), and total tdTomato+ HCs (G). **p < 0.01, ****p < 0.0001. “n” means biological replicates.

To resolve this, we designed EdU labeling and lineage tracing experiments to verify the specific regeneration route. After activating *Cre* recombinase, neonatal *Dkk3* cKO mice were administered EdU to label dividing cells **(Figure 3A)**. No EdU+ SCs were detected in either *Dkk3* cKO or control mice **(Figure 3B)**, which suggests that ectopic HCs may not be generated through mitosis. We performed genetic lineage tracing of *Lgr5*+ progenitors by crossing *Lgr5*^CreER/+^ drivers with Rosa26-tdTomato reporters, tagging both *Lgr5*+ progenitors and their daughter cells during HC generation.

In the next experiment, we used *Lgr5*^CreER/+^*Dkk3*^f/f^Rosa26-td*Tomato* mice for lineage tracing, with *Lgr5*^CreER/+^Rosa26-td*Tomato* mice used as control **(Figure 3C)**. *Dkk3* cKO mice showed a significant increase in tdTomato+ IHCs (at least 8-fold) and OHCs (at least 3-fold) compared to controls **(Figure 3D)**. Additionally, tdTomato+ HCs demonstrated a base-to-apex increasing gradient throughout the cochlea **(Figures 3E-G)**. Collectively, the absence of EdU incorporation combined with robust lineage tracing confirms that *Dkk3* knockout drives HC spontaneous generation via direct trans-differentiation of *Lgr5*+ progenitors.

### Structural and functional characterization of ectopic HC in P7 *Dkk3* cKO mice

**Figure 4:**
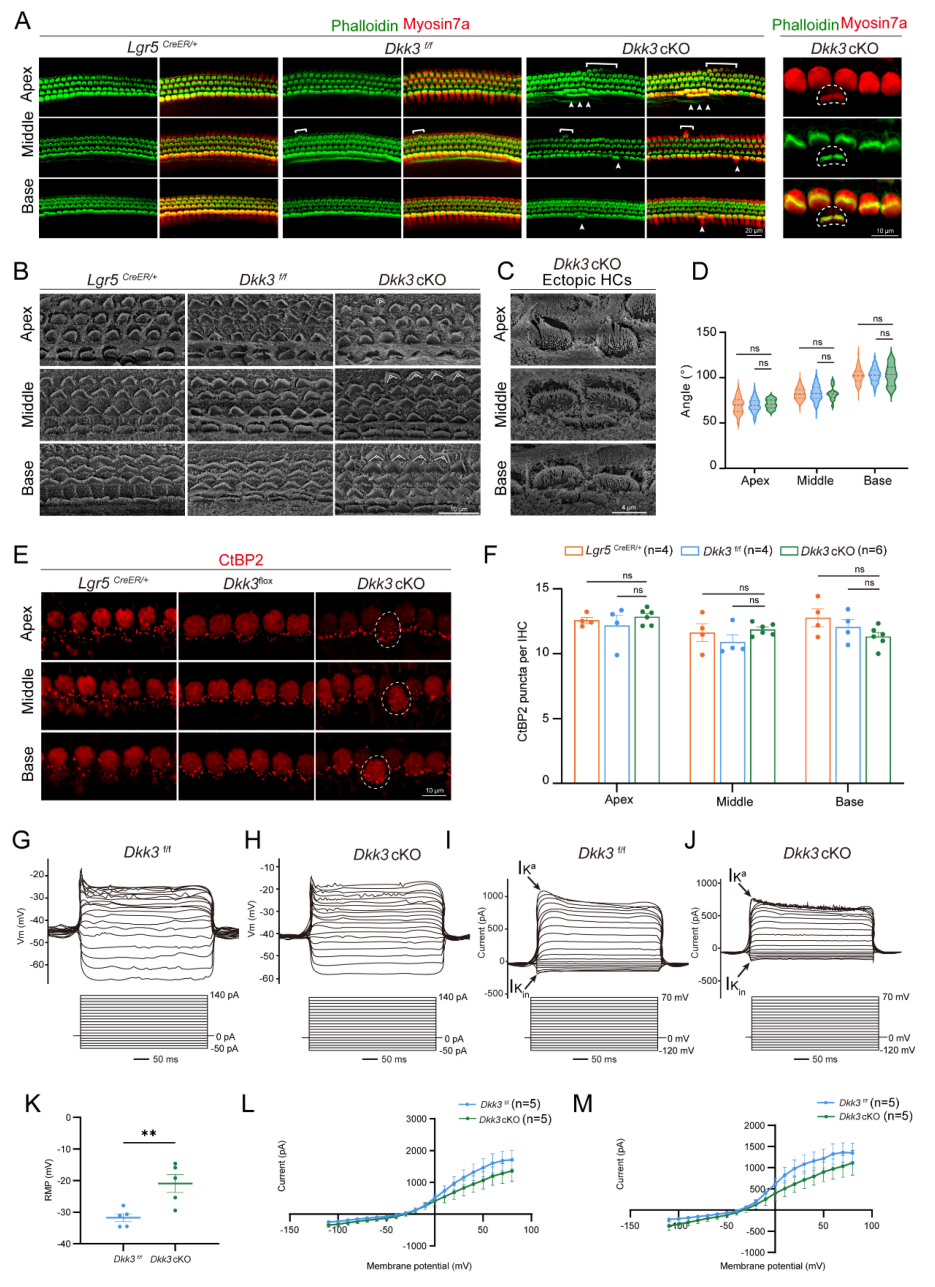
** HC makers staining, hair bundle, and synapse of the P7 ectopic HCs. (A)** Phalloidin staining of hair bundles in P7 cochleae. Arrows indicate ectopic IHCs, and brackets indicate ectopic OHCs. *Lgr5*^CreER/+^ mice and *Dkk3*^f/f^ mice were used as control groups. Scale bar, 20 µm (Right) and 10 µm (Left). **(B-C)** SEM of hair bundles in ectopic HCs across cochlear turns (B) and high-magnification view (C). Scale bars: 10 µm (B), 4 µm (C).** (D)** Quantitative analysis of the angle of ectopic OHCs stereocilia.** (E-F)** CtBP2 staining (E) and quantitative analysis (F) of synapses in IHCs. Dotted white circles indicate ectopic IHC and its Ctbp2+ synapses. Scale bars, 10 µm. *ns*, not significant. **(G-H)** Representative evoked spikes in native OHCs and ectopic OHCs from *Dkk3*^f/f^ (G) and *Dkk3* cKO (H) mice were captured under current-clamp mode, respectively. Step current injection (from -50 to 140 pA, 10 pA per step) elicited spikes and voltage responses that were recorded. **(I-J)** Example of K+ currents of native OHCs and ectopic OHCs from *Dkk3*^f/f^ (I) and *Dkk3* cKO (J) mice recorded in voltage clamp mode, respectively. **(K)** Resting membrane potential (RMP) of native and ectopic OHCs (n = 5). **(L-M)** The I–V plot of the averaged fast component (L) and low component (M) of the K+ currents corresponding to (B). n =5. **p < 0.01. “n” means biological replicates.

To assess the developmental status of ectopic HCs in P7 *Dkk3* cKO cochleae, we examined the molecular, structural, synaptic, and electrophysiological properties. Ectopic HCs expressed the pan-HC marker *Calbindin 1*, while *Prestin* and *Otoferlin* serve as functional markers for mature OHC and IHC, respectively^44^, ^45^. Their expression levels were comparable to those observed in native HCs **(Figure S4A, B)**, confirming subtype-specific molecular maturation comparable to age-matched native controls. Phalloidin staining and scanning electron microscopy (SEM) revealed normally structured stereociliary bundles in ectopic HCs **(Figure 4A-C)**. The V-shaped orientation of stereocilia exhibited a tonotopic gradient along the cochlear axis, consistent with that of age-matched native HCs in the controls **(Figure 4D)**. Subsequently, *CtBP2* staining was used to label the synapses of the IHCs, revealing that the ectopic IHCs exhibited synapse counts comparable to those of native IHC controls** (Figure 4E, F)**.

To evaluate the functional properties of ectopic OHCs, we performed whole-cell patch-clamp recordings and compared their electrophysiological properties to those of native OHCs. In *Dkk3* cKO mice at P7, native OHCs exhibited a higher propensity for action potential generation compared to ectopic OHCs **(Figure 4G, H)**. We further analyzed voltage-gated potassium currents (Iₖ) and identified the presence of fast-inactivating K⁺ currents (Iₖₐ) in ectopic OHCs** (Figure 4I, J)**. Notably, the resting membrane potential (RMP) differed significantly between groups: ectopic OHCs displayed an RMP of –20.92 ± 6.36 mV, whereas native OHCs had a more hyperpolarized RMP of –31.76 ± 2.83 mV** (Figure 4K)**. Additionally, stepwise current injections revealed progressively reduced amplitudes of both the fast and slow components of averaged potassium currents in ectopic OHCs relative to native HCs **(Figure 4L, M)**. Together, these findings demonstrate that *Dkk3* cKO drives the formation of ectopic HCs that are molecularly and structurally differentiated, possessing organized stereocilia and synaptic ribbons, comparable to age-matched controls. These cells also successfully acquire core biophysical machinery, including voltage-gated currents and excitability, while displaying a signature consistent with a nascent developmental state. Accordingly, we designate this specific population as ‘immature HCs’ to facilitate the subsequent single-nucleus transcriptomic analysis.

### Single-nucleus RNA sequencing suggests the fate conversion pathway from SCs to HCs

**Figure 5:**
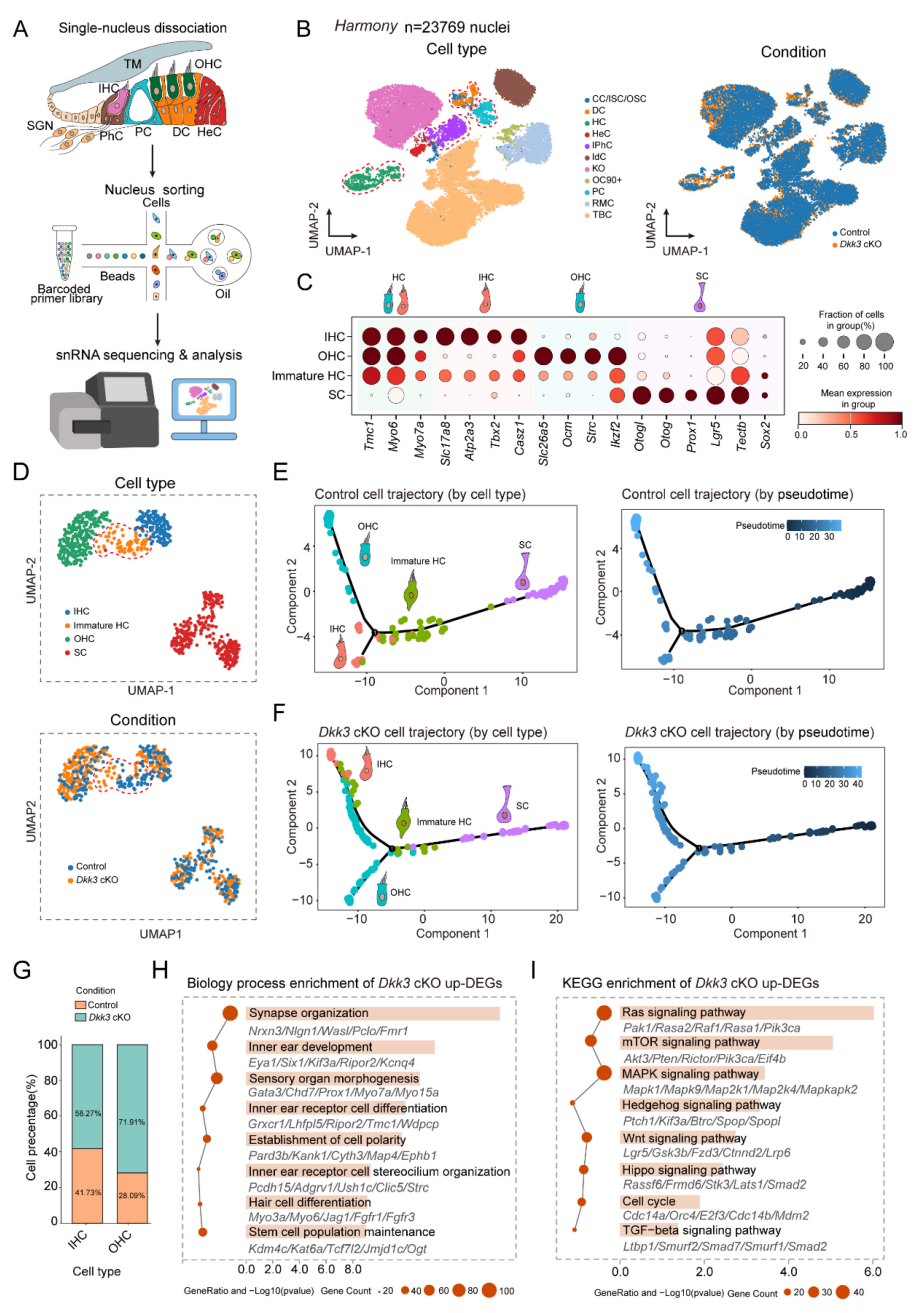
** An snRNA-seq atlas of fate conversion from supporting cells to HCs. (A)** Experimental workflow for snRNA-seq of cochlear sensory epithelium from Control and *Dkk3* cKO mice. **(B)** Integrated transcriptomic landscape of cochlear sensory epithelial cells from Control and *Dkk3* cKO mice, harmonized using the Harmony algorithm. UMAP projection of 23,769 nuclei, annotated by canonical marker genes (CC/ISC/OSC, Claudius Cells/Inner sulcus cell/Outer sulcus cells; OC90+, OC90 positive cells; PC, Pillar cells; HeC, Hensen's cells; IdC, Interdental cells; TBC, Tympanic border cells; RMC, Reissner's membrane cells). The red dashed line indicates DCs, PCs, IPhCs, and HCs for subsequent analysis. The right panel depicts cell embeddings stratified by genotype. **(C)** Expression of canonical markers used to identify SCs, immature HCs, IHCs, and OHCs. **(D)** Integrated UMAP visualization of 443 nuclei from SC and HC lineages across Control and *Dkk3* cKO groups following *Harmony* alignment. The red dashed line indicates immature HC **(E–F)** Pseudotime trajectory analysis illustrating the inferred developmental progression from SCs through immature HC toward IHC and OHC lineages in both Control and *Dkk3* cKO groups. **(G)** Proportion of IHC and OHC in both the Control and *Dkk3* cKO groups. **(H–I)** Functional enrichment analysis of upregulated DEGs in the *Dkk3* cKO group. (H) GO biological processes; (I) KEGG pathway analysis.

To investigate the impact of *Dkk3* cKO in SC on their trans-differentiation into HC, we performed snRNA-seq on auditory sensory epithelial cells isolated from both control and *Dkk3* cKO mice **(Figure 5A)**. After initial quality control, we retained 13,952 high-quality nuclei from control and 17,153 from *Dkk3* cKO samples **(Figure S5A, B)**. Cell type annotation was performed based on established marker genes for known cochlear cell types **(Figure S5C)**, leading to the identification of 11 distinct cell clusters in each group. To mitigate batch effects between conditions while preserving biologically meaningful variation, we systematically evaluated six integration algorithms—*Harmony*, *scVI*, *scANVI*, *Scanorama*, *ComBat*, and *BBKNN* **(Figure 5B; Figure S6A)**. Among these, *Harmony* demonstrated superior performance in effectively removing technical artifacts while retaining true biological variance **(Figures S6B, C)**. We therefore selected *Harmony* for data integration and downstream analyses. Following integration and additional filtration to exclude low-quality cells, a total of 23,769 nuclei (control: 12,750; *Dkk3* cKO: 11,019) were retained for subsequent analysis** (Figure 5B)**.

Given the focus of this study on the trans-differentiation process from SCs to HCs, we isolated *Lgr5*+ SCs (including DCs, PCs, and IPhCs) and HCs from P7 mouse cochleae for re-clustering and cell type annotation. Using well-established marker genes for SCs and HCs, we identified three distinct populations: SCs, mature HCs, and a distinct cluster co-expressing both SC and HC markers, which we designated as “immature HCs”—putatively representing a transitional state during SC-to-HC trans-differentiation** (Figure 5C, D)**. To further elucidate the developmental positioning of this population within the trans-differentiation continuum, we performed cell trajectory analysis. The results revealed a continuous differentiation path from SCs through the immature HC state toward mature IHC and OHC in both control and *Dkk3* cKO groups. The results showed that in the control group and *Dkk3* cKO group, SC continued to differentiate into mature IHC and OHC through immature HC state, and immature HC in the *Dkk*3 cKO group was more inclined towards the end of the differentiation trajectory **(Figure 5E, F)**. Notably, the *Dkk3* cKO group exhibited a 43.82% increase in OHCs and a 16.54 % increase in IHCs compared with controls, suggesting that *Dkk3* cKO in SCs promotes trans-differentiation into HCs** (Figure 5G)**. Subsequent differential expression gene (DEG) analysis between *Dkk3* cKO and control groups identified 2365 upregulated and 88 downregulated genes. GO enrichment analysis indicated that upregulated genes were significantly associated with inner ear development-related biological processes **(Figure 5H)**. KEGG pathway analysis further demonstrated potential enrichment of the Wnt and Hedgehog signaling pathways in the *Dkk3* cKO group **(Figure 5I)**.

To validate these bioinformatic predictions and comprehensively assess the molecular landscape triggered by *Dkk3* knockout, we performed RT-qPCR quantification on basilar membrane (BM) tissues isolated from P7 mice. This targeted validation screened a broad spectrum of signaling cascades identified in our pathway enrichment analysis. Consistent with the snRNA-seq profiles, *Dkk3* cKO cochleae exhibited significant upregulation of key genes associated with the Wnt (*Lgr5, Tcf7l2*) and Hedgehog (*Fbxw11, Smurf1*) pathways, providing a direct mechanistic basis for the observed phenotype (**Figure S7D, E**). Furthermore, we validated significant alterations in Ras, mTOR, MAPK, Hippo, and TGF-β signaling pathways (**Figure S7A, B, C, F, G**). We also observed the upregulation of cell cycle regulators (*E2f3, Mdm2*) (**Figure S7H**). In the absence of EdU incorporation, this distinct molecular signature suggests that *Dkk3* knockout induces a state of cellular plasticity characterized by the reactivation of developmental and cell-cycle machinery, thereby facilitating direct trans-differentiation rather than proliferative expansion.

### Regenerated ectopic HCs in *Dkk3* cKO mice could survive until adulthood

**Figure 6:**
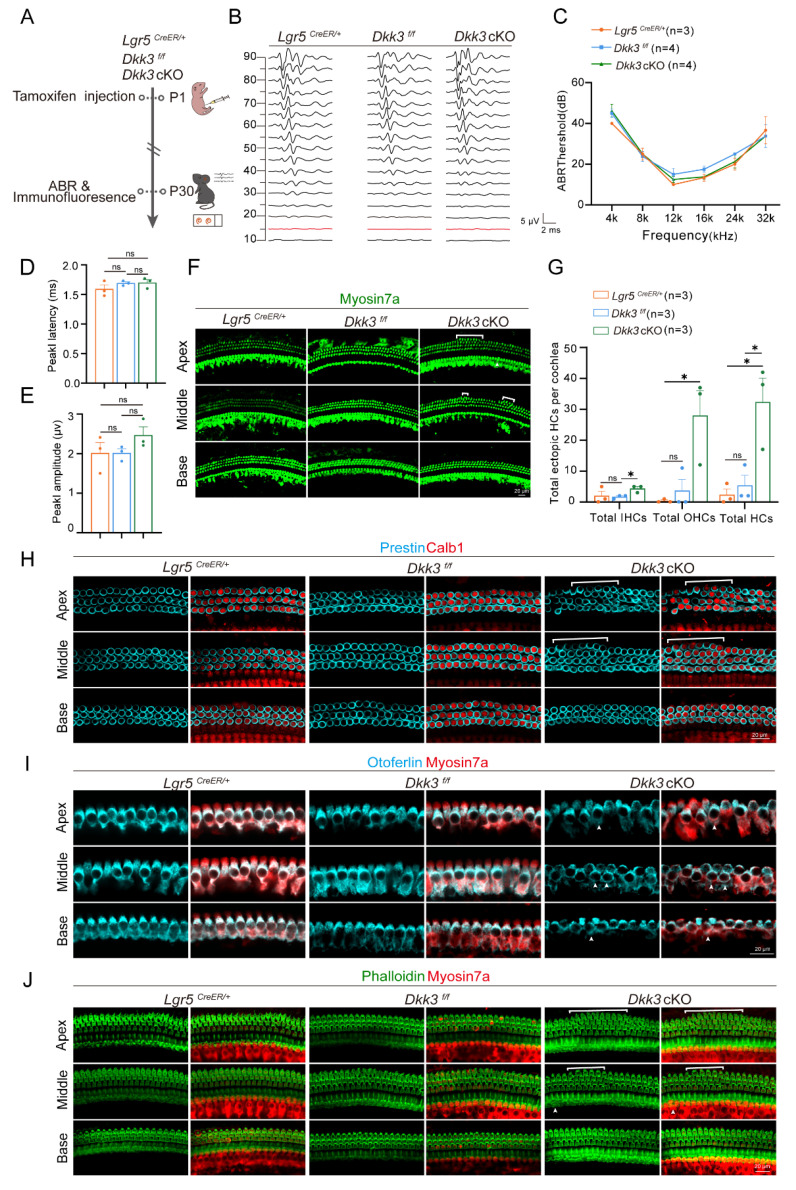
** Characteristics of ectopic HCs in P30* Dkk3* cKO mice. (A)** Flowchart of the ABR testing for *Dkk3* cKO mice. **(B)** Representative ABR waveforms at 16 kHz. Red trace indicates threshold. Scale bar applies to all traces. The red trace indicates the threshold. **(C)** ABR thresholds across frequencies (4–32 kHz) in *Dkk3* cKO and control mice. **(D-E)** Peak I latency (D) and amplitude (E) of the ABR wave at 16 kHz, 90 dB between *Dkk3* cKO mice and control mice. **(F)** Confocal images of the P30 cochlea stained with Myosin7a. Arrows indicate ectopic IHCs and brackets indicate ectopic OHCs. Scale bars, 20 µm. **(G)** Quantitative analysis of ectopic IHCs, ectopic OHCs, and total ectopic HCs per cochlea in P30 *Dkk3* cKO and control mice. *p < 0.05, and ns, not significant. **(H)** Representative confocal images of Calb1 and Prestin in cochlear turns between P30 *Dkk3* cKO mice and controls. Scale bars, 20 µm.** (I)** Representative confocal images of Myosin7a and Otoferlin in cochlear turns between P30 *Dkk3* cKO mice and controls. Scale bars, 20 µm. **(J)** The hair bundles of HCs were stained using phalloidin in P30 *Dkk3* cKO mice. Scale bar, 20 µm.

To further evaluate the functional consequences of *Dkk3* cKO, we assessed auditory function in P30 *Dkk3* cKO mice using auditory brainstem response (ABR) measurements. Hearing thresholds across frequencies from 4 to 32 kHz were comparable to those of control groups **(Figure 6A–C)**. Moreover, neither the latency nor the amplitude of peak I differed significantly between groups **(Figure 6D, E)**.

We next confirmed the structural persistence of these ectopic cells via immunofluorescence. Although the absolute number of ectopic HCs decreased compared to the neonatal peak at P7, which likely reflects a process of developmental pruning, a substantial population survived into adulthood **(Figure 6F, G)**. Importantly, these persistent cells maintained a stable mature phenotype. They continued to express specific markers for OHCs and IHCs **(Figure 6H, I)** and retained structurally intact stereociliary bundles **(Figure 6J)**. These findings indicate that *Dkk3* knockout initiates a reprogramming signal to support long-term molecular and morphological stability without inducing deleterious effects on the native auditory organ.

## Discussion

SNHL, resulting from HC loss due to noise exposure, ototoxic drugs, or aging, remains irreversible in mammals due to the inability of the cochlea to reactivate the intrinsic regenerative process. *Lgr5*+ progenitors have emerged as promising targets for HC regeneration strategies. Based on the existing research, some progress has been made, including overexpression of key transcription factors^46^, ^47^, nerve growth factors^48^, ^49^, cochlear secretory factors^50^, and signaling pathway regulators^51^, ^52^. However, generating fully mature and functional HCs remains challenging.

In this study, we identified *Dkk3* as a critical negative regulator of cochlear *Lgr5+* progenitor plasticity. By integrating transcriptomic analysis of *Lgr5*+ progenitors across different spatial, injury-response, and lineage-specific contexts, we identified *Dkk3* as one of 14 candidate genes linked to regenerative potential. Previous research suggests *Dkk3* is essential for regulating the fate and differentiation of muscle cells^53^, ^54^. *Dkk3* inhibits muscle stem cell differentiation and impairs generation* in vivo*, yet its knockout improves muscle repair in obese models^55^. The loss of *Dkk3* enhances induced pluripotent stem cell (iPSC) generation *in vitro* and drives proliferation in hepatocytes and *Lgr5*+ liver progenitors in mice^56^. Consistent with these roles, we found that *Dkk3* KD significantly increased the sphere-forming efficiency of *Lgr5*+ progenitors *in vitro*. While the seeding density required for these primary cell assays (2,000 cells/well) precludes strict clonal analysis, the robust increase in sphere number indicates a significant enhancement of population-level regenerative capacity and survival.

*Dkk3* cKO in *Lgr5*+ progenitors robustly stimulated the generation of ectopic IHC and OHC across all cochlear turns *in vivo*. Lineage tracing and EdU assays confirmed that these ectopic HCs arise via direct trans-differentiation rather than mitotic generation. Although *in vitro* results suggested a pro-proliferative role for *Dkk3* knockdown, the absence of EdU+ SCs *in vivo* indicates that the regenerative outcome is context-dependent, likely influenced by the native cochlear microenvironment. Our findings establish *Dkk3* knockout as a potent mechanism to promote the innate regenerative capacity of cochlear SC, driving robust HC generation through direct trans-differentiation.

Previous efforts to regenerate HCs have primarily relied on overexpressing transcription factors like *Atoh1*, either alone or in combination. While these approaches can induce HC fate, the resulting cells typically suffer from transient survival, disorganized stereocilia, incomplete marker expression, and an inability to functionally integrate, often leaving auditory deficits unresolved^57^, ^58^,^59^, ^60^. In contrast, our *Dkk3* cKO model resolves several of these limitations. The ectopic HC in *Dkk3* cKO mice exhibited remarkable molecular and structural maturity, expressing key markers such as *Prestin* and *Otoferlin*, forming organized stereociliary bundles, and establishing synaptic connections comparable to native HC. Importantly, these newly formed HC persist into adulthood without compromising auditory function, as evidenced by normal ABR thresholds and wave I parameters. These results show that the mammalian cochlea can sustain ectopic sensory cells over the long term, provided the relevant inhibitory constraints are removed.

Notably, snRNA-seq analyses revealed that *Dkk3* does not act in isolation but rather functions as a master suppressor. Its knockout may trigger the coordinated activation of a broad pro-regenerative network, including Ras, Wnt, Hedgehog, mTOR, MAPK, and Hippo signaling pathways^52^, ^61^-^63^. Furthermore, our targeted validation highlighted a significant upregulation of Ras expression. Considering the well-established Ras-MAPK signaling cascade^64^, this upregulation is consistent with the activation of MAPK pathways, which are known to support SC proliferation and HC survival^65^-^67^. While each of these pathways has been individually implicated in HC development, their simultaneous upregulation creates a highly permissive landscape that facilitates both fate conversion and subsequent differentiation processes, such as cell polarity establishment and synaptogenesis. This multi-pathway activation likely explains the superior structural organization and differentiation status observed in our model compared to single-pathway interventions.

Although these reprogrammed HC achieve structural stability, they display a nascent electrophysiological profile, characterized by elevated resting membrane potentials and immature potassium currents. However, the fact that these cells successfully integrate into the sensory epithelium and persist long-term provides critical proof-of-concept that the physical and metabolic support systems of the adult cochlea are sufficient to maintain spontaneously regenerated HCs.

Looking forward, the multi-pathway mechanism activated by *Dkk3* knockout provides a strong rationale for combinatorial therapeutic strategies. For instance, coupling *Dkk3* suppression with* Atoh1* overexpression may synergize to enhance both the initiation of HC fate and long-term functional maturation^19^. Similarly, supplementing *Dkk3* inhibition with neurotrophic factors^48^, ^68^ or ion channel modulators^69^ could further promote synaptic integration and electrophysiological maturity. Our study establishes *Dkk3* inhibition not only as a novel target for inducing direct cellular reprogramming but also as a foundational framework for developing durable, multi-modal therapies for functional hearing restoration.

## Materials and Methods

### Mice and genotyping

*Dkk3*^flox^ mice (Cyagen, S-CKO-11394) and *Lgr5*^CreER/+^mice (Jackson Laboratory, stock 008875) were maintained under a specific pathogen-free (SPF) barrier system. The mouse tail tips used for genotyping were digested in 90 μl 50 mM NaOH at 98 °C for 1 hour, and 10 μL Tris-HCl (pH = 8) was added to terminate digestion. Primer sequences for PCR amplification are detailed in **Supplemental Table S2**. All animal protocols were approved by the Southeast University Institutional Animal Care and Use Committee (No. 20210302031) and conducted under NIH guidelines for humane animal experimentation, with sample sizes statistically optimized to ensure rigor while minimizing animal usage.

### *Dkk3* cKO induction and proliferation assessment

To induce cKO of *Dkk3* in *Lgr5*+ progenitors, both *Dkk3* cKO and control mice received *i.p.* injections of tamoxifen (Sigma, T5648; 0.075 mg/g body weight) at P1 to activate *Cre* recombinase. To assess SC proliferative activity,* Lgr5*^CreER/+^ and *Dkk3*^f/f^ mice received *i.p.* injections of EdU (Sigma, 900584; 0.05 mg/g body weight) from P3 to P5. Proliferating EdU+ SCs were labeled and detected using the Click-iT^TM^ EdU imaging kit (Invitrogen, C10337).

### Frozen section preparation

Temporal bones from P2 mice were dissected in PBS and fixed in 4% PFA overnight at 4 °C. After PBS rinsing, samples were immersed in 15%, 20%, and 30% sucrose solutions, followed by sucrose: OCT mixtures (1:1, 3:7, 1:9), under vacuum infiltration for 1 hour, and incubated overnight at 4 °C for each step. Tissues were then embedded in pure OCT under vacuum for 1 hour and incubated overnight at 4 °C. Specimens were oriented with the oval or round window facing downward, frozen at –20 °C for 1 hour, and stored at –80 °C. Coronal sections (14 μm) were obtained at –23 °C using a freezing microtome (RWD, FS800) and mounted on adhesive slides (Citotest, 188105).

### RNAscope *in situ* hybridization

Cryosections were dried at 60°C for 30 minutes, fixed in pre-cooled 10% NBF for 15 minutes, and dehydrated through an ethanol series. Endogenous peroxidase activity was blocked with hydrogen peroxide (10 minutes, room temperature). Antigen retrieval was performed in boiling RNAscope® Target Retrieval Reagent for 5 minutes, followed by post-fixation in 10% NBF (30 minutes, room temperature). Protease III treatment was conducted at 40 °C for 30 minutes. Sections were hybridized with a *Dkk3* (C1) probe (ACDBio, Catalog No. 400931) at 40 °C for 2 hours. Signals were amplified using AMP1–3 reagents and Opal 520 fluorophore (Opal, ASOP520), with HRP blocking between steps. Finally, sections were counterstained with DAPI and mounted with DAKO fluorescent medium. The above reagents are sourced from the RNAscope *in situ* hybridization kit (Advanced Cell Diagnostics, 323110; 323180).

### Immunofluorescence

Following fixation and decalcification, mouse cochleae were micro-dissected into apical, medial, and basal regions in ice-cold PBS. Tissue sections were blocked in blocking medium supplemented with 5% donkey serum, 1% bovine serum albumin, 0.5% Triton X-100, and 0.02% sodium azide for 2 hours. After primary antibody incubation at 4°C overnight, the species-matched Alexa fluor-conjugated secondary antibodies were incubated for 1 hour. Samples were mounted using DAKO fluorescent mounting medium (Dako, S3023) and stored in the dark at 4 °C. The antibodies used are detailed in **Supplementary Table 3**.

### Scanning electron microscopy

Cochlear specimens underwent sequential processing beginning with 2.5% glutaraldehyde fixation (4 °C, 24 hours) and 0.5M EDTA decalcification (25 °C, 90 min). Ethanol dehydration gradients (50%, 70%, 90%, 100%; 15 min per step) preceded critical point drying (Leica CPD300) with liquid CO₂. Specimen preparation included conductive mounting (carbon adhesive) and 30-100s Au sputter-coating (Quorum Q150T ES plus). Ultramicrostructural analysis was performed using field-emission SEM (FEI Nova Nano 450) under high vacuum conditions.

### Western blotting

Tissue lysates were prepared in RIPA buffer (Epizyme, PC101) supplemented with protease inhibitor cocktail (Roche, 04693132001). Protein quantification via BCA assay (Epizyme; E112-01) standardized sample loading (20 μg/lane) for electrophoretic separation on 10% SDS-PAGE gels (Epizyme; E303-01) at 120V for 100 min. Following wet-transfer to PVDF membranes (Millipore; IPVH00010), blocking was conducted with 5% skim milk (Beyotime; P0216) in TBST for 2 hours at room temperature. Membranes underwent sequential incubations with primary antibodies and HRP-conjugated secondaries. Chemiluminescent detection employed an ECL kit (Vazyme; E422) with image acquisition on a Tanon 2500R system. The antibodies used are detailed in **Supplementary Table 3**.

### Cell culture and transfection

HEI-OC1 cells were cultured in DMEM (Gibco; 11995500) containing 10% FBS (Pansera; P30-2602) and 100 μg/mL ampicillin (Beyotime; ST008) under standard conditions (37 °C, 10% CO₂). Cells were digested using 0.25% trypsin-EDTA (Gibco, 25200072) and planted into the 6-well plate (Greiner, 657160). 25 pmol siRNA (Shanghai Gene Pharma) was transfected into cells by using Lipofectamine RNAiMAX (Invitrogen, 13778075) in Opti-MEM Reduced Serum Medium (Gibco, 31985062). siRNA sequences with no homology to target cells were used as negative controls (NC). Shanghai Gene Pharma synthesized siRNA, and the siRNA sequences are listed in **Supplemental Table 4**.

### RNA extraction and RT-PCR

Total RNA was isolated from cochleae or HEI-OC1 cells using TRIzol™ reagent (Invitrogen, 15596026). Following phase separation with 200 μL chloroform (Sinopharm Chemical Reagent Co., Ltd., 10006818), RNA was precipitated with 500 μL isopropanol (Sinopharm Chemical Reagent Co., Ltd., 40064360) and washed with 75% ethanol. Purified RNA was resuspended in DEPC-treated water, and concentration/purity was quantified using a Nano-500 spectrophotometer (Allsheng, nano-500). Reverse transcription was conducted with 1 μg total RNA using the RevertAid First Strand cDNA Synthesis Kit (Thermo Fisher Scientific, K1622). RT-qPCR analysis was performed using the QuantStudio™ 3 Real-Time PCR System (Thermo Fisher Scientific) and FastStart Universal SYBR Green Master Mix (Roche, 4913914001). And the primers used are listed in **Supplemental Table 5**.

### Flow cytometry

*Lgr5*+ progenitors were isolated from P1 *Lgr5*-EGFP-IRES-creERT2 transgenic mice (n≈30). Cochleae were micro-dissected in ice-cold PBS (1×, Gibco, 10010023) and enzymatically dissociated using 150 μL 0.25% trypsin (Gibco, 15050065) at 37 °C for 10 min. Mechanical dissociation was completed by pipetting up to 300 μL trypsin inhibitor (Worthington Biochemical, LS003570), followed by filtration through a 40-μm cell strainer (BD Falcon, 352340). *Lgr5*+ progenitor populations were isolated by FACS (BD, FACSAria III) based on GFP fluorescence intensity.

### Sphere assay

FACS-sorted *Lgr5*+ cells were cultured in ultra-low attachment 96-well plates at a density of 2000 cells per well (Corning, 3474) using advanced-DMEM/F12 medium (Gibco, 12634010) supplemented with 1% N-2 (Gibco, 1750248), 2% B-27 (Gibco, 17504044), 20 ng/mL EGF (Sigma-Aldrich, E9644), 50 ng/mL IGF-I (Sigma-Aldrich, I8779), 20 ng/mL heparan sulfate (Sigma-Aldrich, H4777), 10 ng/mL FGF2 (Sigma-Aldrich, F0291), and 0.1% ampicillin (Beyotime, ST008). For gene knockdown, 1 pmol siRNA targeting candidate genes (per well) was transfected into *Lgr5*+ cells on day 1 of culture by using RNAiMAX (Invitrogen, 13778075). After 5 days, organoid spheres derived from *Lgr5*+ progenitors were imaged in brightfield using an inverted fluorescence microscope (Zeiss). Sphere diameter and count were quantified from whole-well images using ImageJ.

### Auditory brainstem response measurement in adult mice

Mice were anesthetized with intraperitoneal injection of amobarbital. Subdermal needle electrodes were inserted at the top of the skull and the mastoid region of both ears. Acoustic stimuli (tone bursts from 4 to 32 kHz) were delivered through a tube speaker placed 10 cm from the test ear. Stimulus intensity was decreased in 5-dB steps from 90 to 10 dB SPL. Auditory evoked potentials were filtered, amplified, and averaged (256 repetitions per intensity). Threshold was defined as the lowest intensity level at which a reproducible wave I pattern was visible. The tested mice were placed on a heating pad to maintain body temperature.

### Electrophysiological recording

Electrophysiological recordings from cochlear tissues were performed as previously described^70^. Briefly, we dissected the apical turn of the cochlea from P7–8 *Dkk3*^flox/flox^ or *Dkk3*-cKO mice of both sexes. Whole-cell patch-clamp recordings were conducted on native HCs and ectopic HCs under identical conditions. The tissues were embedded and continuously perfused with ice-cold, sterile HEPES-buffered artificial cerebrospinal fluid (ACSF). The ACSF composition was as follows (in mM): 115 NaCl, 6 KCl, 1.3 NaH_2_PO_4_, 11.2 NaHCO_3_, 11 D-glucose, 1.3 MgCl_2_, and 1.3 CaCl_2_^71^. Cochlear cells were visualized using an inverted microscope equipped with differential interference contrast optics and a 60×/1.00 NA water-immersion objective. Native and ectopic HCs were distinguished based on their cellular arrangement and ciliary morphology under 60× magnification. Recordings were acquired using an Olympus BX51WI microscope and Axon 7500B amplifiers. Patch pipettes with resistances of 10–15 MΩ were filled with an internal solution containing (in mM): 135 KCl, 3.5 MgCl_2_, 0.1 CaCl_2_, 5 EGTA-KOH, 5 HEPES-KOH, 2.5 Na_2_ATP (pH 7.35, 325 mOsm). During recordings, both native HCs and HC-like cells were held at a membrane potential of –60 mV. Signals were low-pass filtered at 2 kHz, and data were analyzed using Clampfit 10.2 and GraphPad Prism 9.

### 10× chromium snRNA-seq

snRNA-seq was performed on cochlear tissue isolated from *Lgr5*^CreER/+^ (control) and *Lgr5*^CreER/+^ *Dkk3* ^f/f^ (*Dkk3* cKO) mice at P7, following tamoxifen administration at P1. For each genotype, cochleae from 28 individuals were pooled to generate a single biological replicate. Nuclei were isolated and resuspended at a concentration of 700–1200 nuclei/μl for use with the 10× Genomics Chromium Next GEM Single Cell 3ʹ Reagent Kits v3.1 (Catalog No. 1000268), following the manufacturer’s protocol. Libraries were sequenced on an Illumina NovaSeq 6000 platform (OE Biotech Co., Ltd). Reads were aligned to the mm10_3.0.0 reference genome, and gene expression matrices were generated and quantified using the 10× Genomics Cell Ranger pipeline (v3.8.0.1) with default parameters. The resulting filtered count matrix was used for all subsequent analyses.

### SnRNA-seq data quality control and cell type annotation

Single-cell data analysis was conducted using R (v4.1.3) with Seurat (v4.3.0) and Python (v3.9) with Scanpy (v1.10.3) to enable integrated cross-platform analysis. Initial quality control of the raw gene-barcode matrices derived from 10× Genomics sequencing was performed using Scanpy. Cells were filtered based on the following thresholds: those with fewer than 500 detected genes, more than 5,000 UMIs, or mitochondrial gene content exceeding 5% were classified as low quality and excluded. After filtering, 12,750 high-quality cells from the control group and 11,019 from the *Dkk3* cKO group were retained for downstream analysis. Normalization and log-transformation were applied using standard Scanpy workflows. Highly variable genes were identified using the highly_variable_genes function without imposing an upper bound. Dimensionality reduction was followed by unsupervised clustering using the Leiden algorithm. Resulting clusters were visualized in two dimensions with UMAP. Cell types were annotated based on established marker genes previously reported in the murine cochlea^40^, ^72^.

### Batch effect correction and integration analysis

snRNA-seq data were processed using Scanpy (v1.10.3) following established preprocessing workflows, including quality control filtering, normalization of the cell–gene count matrix, and incorporation of sample metadata^73^. Dataset integration was carried out with *scVI*-tools (single-cell Variational Inference; v1.1.2)^74^. To balance biological conservation and batch-effect removal, we systematically evaluated multiple integration algorithms using the single-cell integration benchmarking framework scIB^75^. Methods tested included *BBKNN*, *Scanorama*,* Harmony*, *ComBat*, *scVI*, and* scANVI*, each implemented under default settings as recommended by the original authors (see GitHub repository for code details). We evaluated the integration methods using the scIB framework, scoring them on both batch effect removal and the preservation of biological variance. Harmony emerged as the top-performing method and was therefore chosen for all subsequent downstream steps.

### Gene ontology enrichment analysis for RNA-seq

DEGs were identified across three comparative groups: ALPs and BLPs, *Lgr5*+ progenitors, and *Lgr5*-SCs^22^-^24^. DEGs were defined using a uniform threshold (| log2(fold change) |> 1.0, *p* < 0.05) and visualized via Venn diagrams generated with the R package *ggvenn*. GO enrichment analysis was performed using *clusterProfiler* (v4.6.0) by using the R package, and the statistical significance threshold level for the GO annotation analysis was *p* < 0.05.

### Cell trajectory analysis

To investigate SC to HC trans-differentiation, *Lgr5*-high SCs (including pillar cells, Deiters' cells, and inner phalangeal cells) and HCs from P7 developmental stages were subclustered after dimensionality reduction. 283 high-quality cells were obtained from the control group, and 443 were obtained from the *Dkk3* cKO group for subsequent analysis. Following integration using *Harmony*, we annotated cell identities into three subtypes: SCs, HCs, and a distinct SC–HC intermediate population. The co-expression of canonical SC and HC markers in the SC–HC group suggests that these cells may represent a transitional state during SC-to-HC differentiation.

To investigate this hypothesis, we performed pseudotemporal trajectory analysis using *Monocle2* (v2.22.0). Data from HCs, SCs, and SC–HC cells from both control and *Dkk3* cKO groups were converted to loom format and imported into R. We constructed a CellDataSet object using the newCellDataSet function, retaining genes detected in at least five cell types. Differential gene expression testing was carried out with the differentialGeneTest function, and genes with q-values < 0.001 were selected for trajectory reconstruction. Dimensionality reduction was performed using the reduceDimension function (max_components = 2, method = “DDRTree”). Cells were ordered along the inferred trajectory using orderCells, and results were visualized with plot_cell_trajectory.

### GO and KEGG enrichment for snRNA-seq

DEG analysis was performed between the control and *Dkk3* cKO groups using DESeq2 (v1.44.0) in combination with zingeR (v0.1.0) to model cell-level weights, accounting for dropout characteristics inherent in snRNA-seq data^76^, ^77^. Genes were considered differentially expressed based on a uniform threshold of |log₂FC| ≥ 0.4 and an adjusted *p*-value < 0.05. Results were summarized and visualized using Venn diagrams generated with the R package *ggvenn*. GO and KEGG enrichment analysis was conducted using *clusterProfiler* (v 4.6.0), with terms considered significantly enriched at a threshold of *p* < 0.05.

### Statistical analysis

Experimental data were derived from ≥3 independent biological replicates, presented as mean ± standard error of mean. Statistical significance was determined by two-tailed unpaired Student's t-tests (GraphPad Prism v10.0.1). *p*-value < 0.05 was considered statistically significant.

## Supplementary Material

Supplementary figures and tables.

## Data Availability

The data that support the findings of this study are available from the corresponding author upon reasonable request.
