# Supplementary material for: Conditional knockout of Dkk3 drives Lgr5+ progenitor reprogramming into hair cells in the mouse cochlea
Source: Theranostics. 2026 May 18;16(12):6911–27. doi: 10.7150/thno.131808 (PMC13232479; doi:10.7150/thno.131808)
Supplement: Supplementary file 1 — Supplementary figures and tables. [file thnov16p6911s1.pdf]

Supporting figures

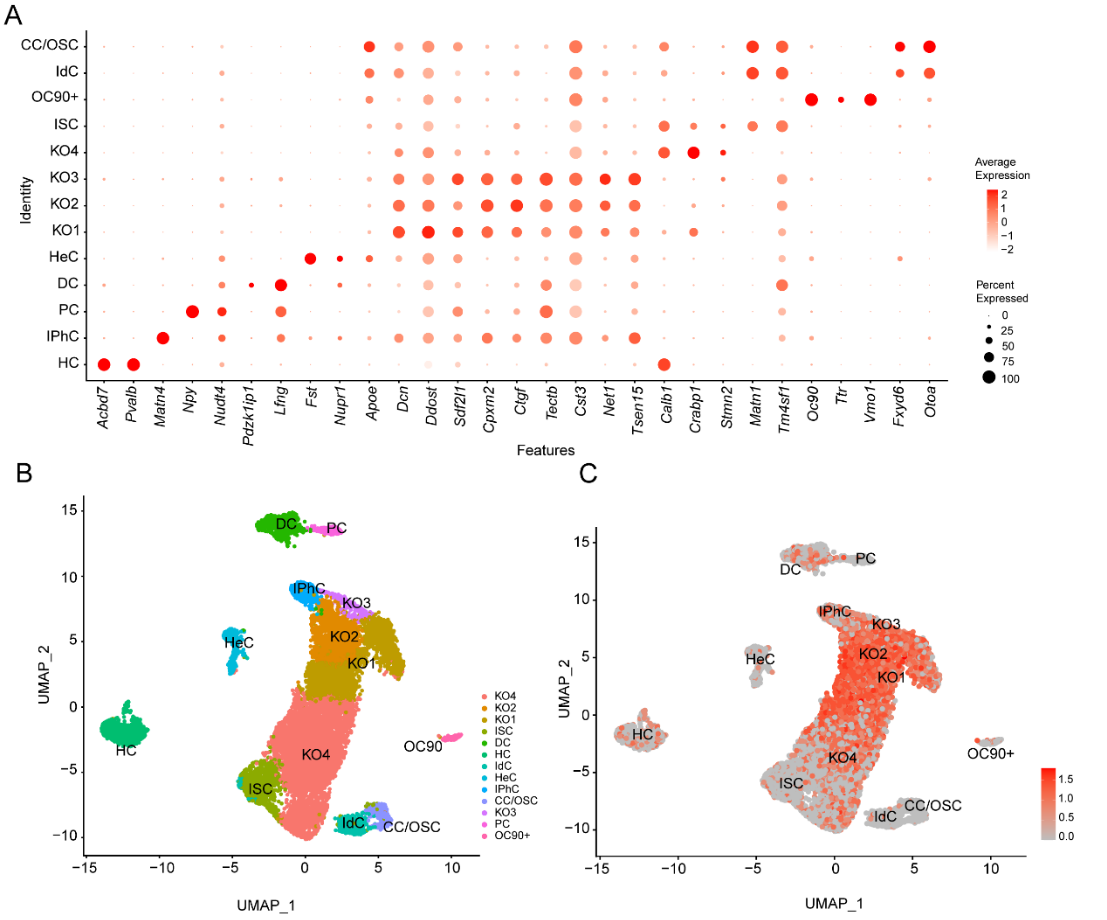

Figure S1. Single-cell transcriptomic analysis reveals the expression pattern of *Dkk3* in the P1 mice cochlea. (A-B) Dot plot (A) and UMAP plot (B) showing 12 identified clusters in the P1 mice cochlea. **(C)** The feature plot highlights the *Dkk*3 gene that was distributed in identified clusters in the P1 mice cochlea. KO1/2/3/4, Kölliker's organ cells 1/2/3/4; ISC, Inner sulcus cells; DC, Deiters' cells; HC, Hair cell; IdC, Interdental cells; HeC, Hensen's cells; IPh, Inner phalangeal cells; CC/OSC, Claudius Cells/outer Sulcus Cells; PC, Pillar cells; OC90+, OC90 positive cells.

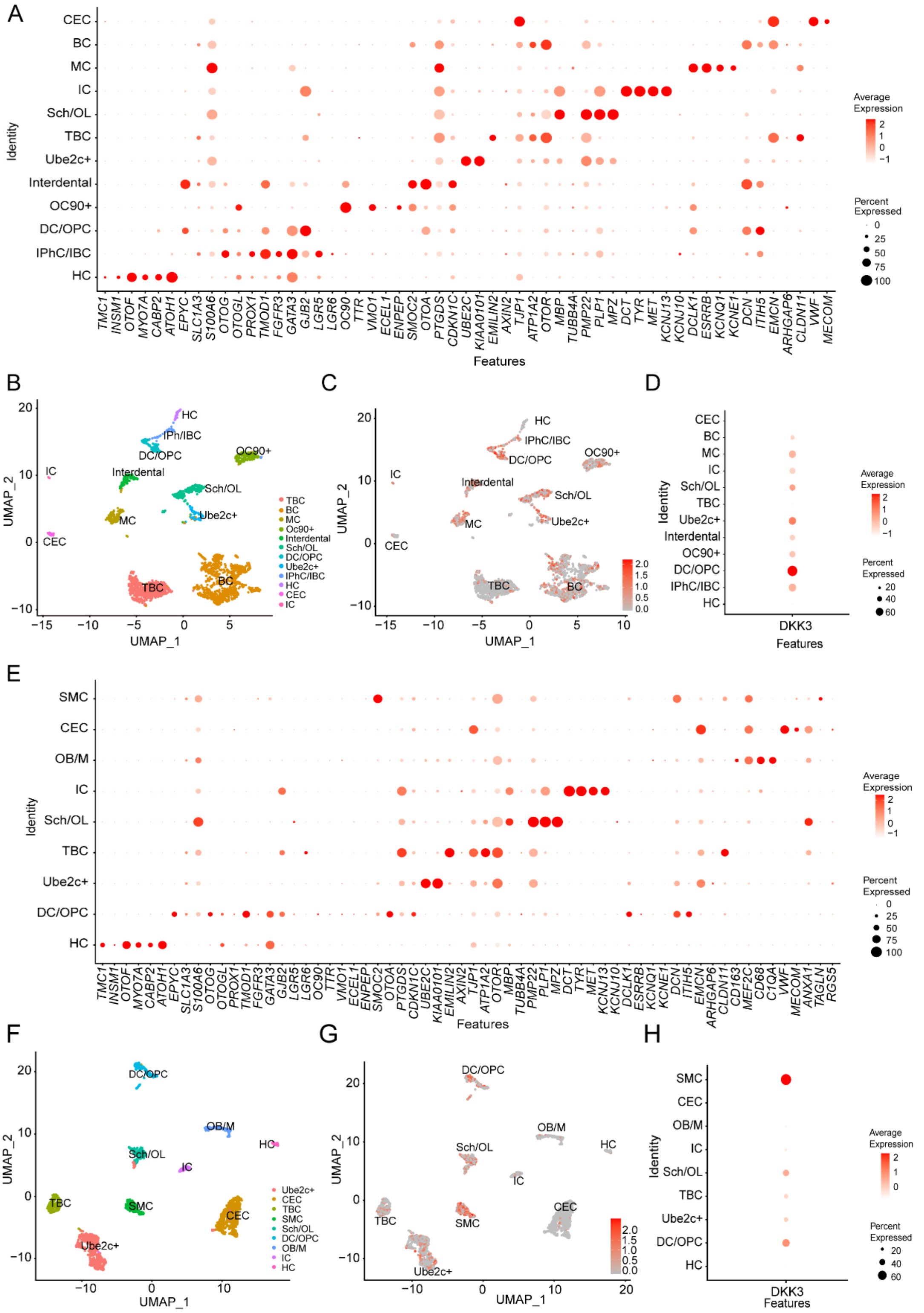

Figure S2. Single-cell transcriptomic analysis reveals the expression pattern of *DKK3* human fetal cochlea. (A-B) Dot plot (A) and UMAP plot (B) showing 12 identified clusters in the 15-week human cochlea. **(C-D)** Feature plot (C) and dot plot (D) highlight the *DKK3* gene that was distributed in identified clusters in the 15-week

human cochlea. **(E-F)** Dot plot (E) and UMAP plot (F) showing 9 identified clusters in the 17-week human cochlea. **(G-H)** Feature plot (G) and dot plot (H) highlight the *DKK3* gene, which was distributed in identified clusters in the 17-week human cochlea. TBC, Tympanic border cells; BC, Basal stria cells; MC, Marginal stria cells; Sch/OL, Schwann cell/ Oligodendrocyte; DC/OPC, Deiters' cells/ outer pillar cells; Ube2c+, Ube2c positive cells; IPhC/IBC, Inner phalangeal cells/ Inner border cells; CEC, Capillary endothelial cells; IC, Intermediate stria cells; SMC, Smooth muscle cells; OB/M, Osteoblast/Macrophage.

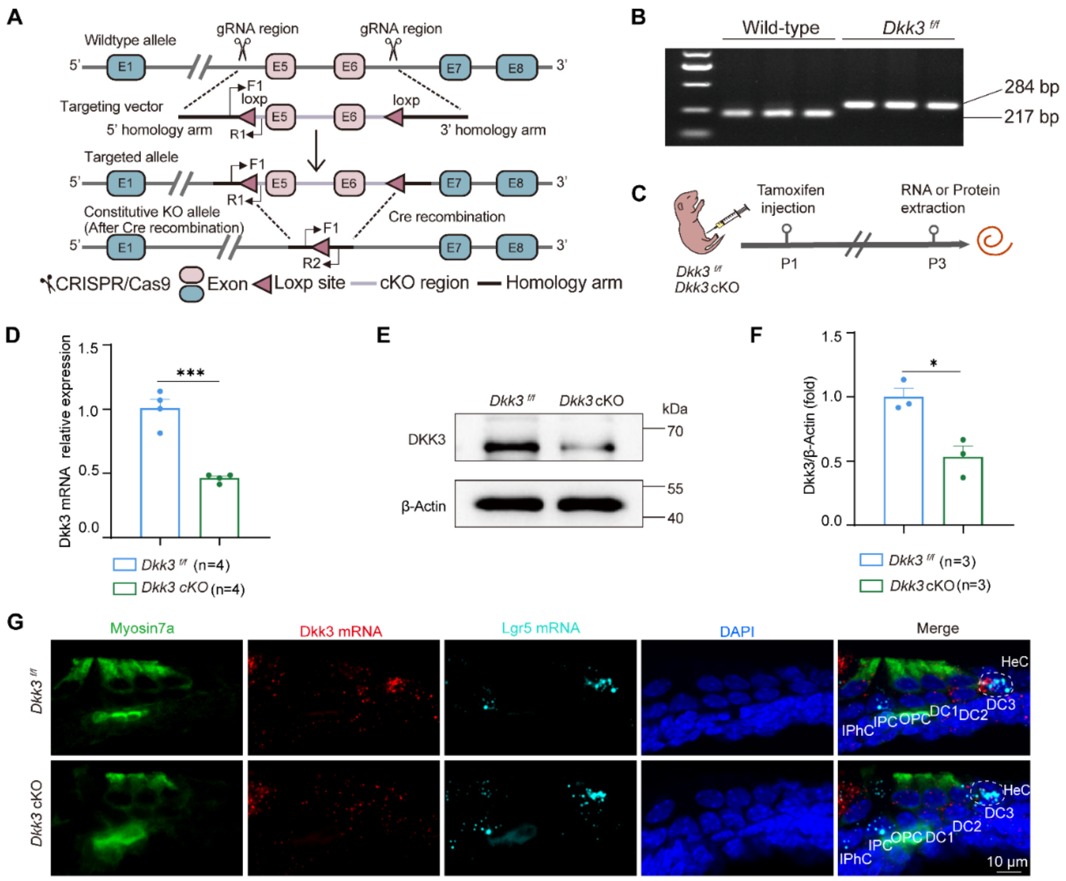

Figure S3. Conditional knockout validation in the *Dkk3* cKO mouse cochlea. (A) Construction strategy of *Dkk3*$^{flox/+}$ mice. **(B)** Genotyping of *Dkk3*$^{f/f}$ mice. **(C)** Flow chart of the *Dkk3* expression pattern study in *Dkk3* cKO mice cochlea. **(D-F)** The Dkk3 expression in *Dkk3* cKO mice was detected by RT-qPCR (D) and western blotting (E); the quantitative analysis of western blotting is shown in (F). **(G)** RNAscope *in situ* hybridization of *Dkk3* and *Lgr5* in *Dkk3* cKO mice and *Dkk3*$^{f/f}$ cochlea. $*p < 0.05$, $***p < 0.001$. "n" means biological replicates.

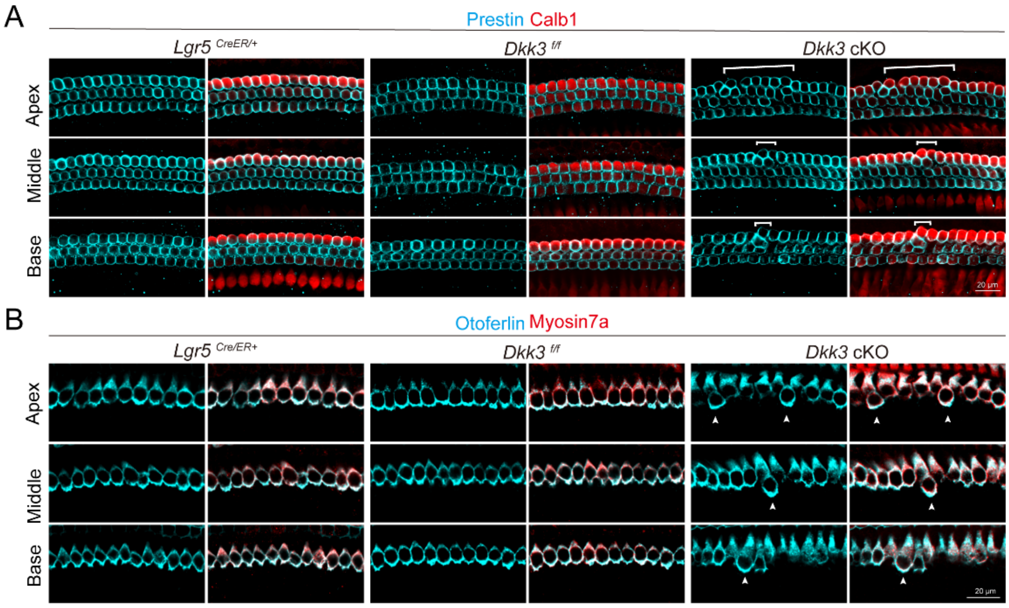

Figure S4. HC makers staining of the P7 ectopic HCs.

(A) Representative confocal images of Calb1 and Prestin (an OHC maker) in the three turns of cochleae between *Dkk3* cKO mice and controls. Scale bars, 20 µm. **(B)** Representative confocal images of Myosin7a and Otoferlin (an IHC maker) in the three turns of cochleae between *Dkk3* cKO mice and controls.

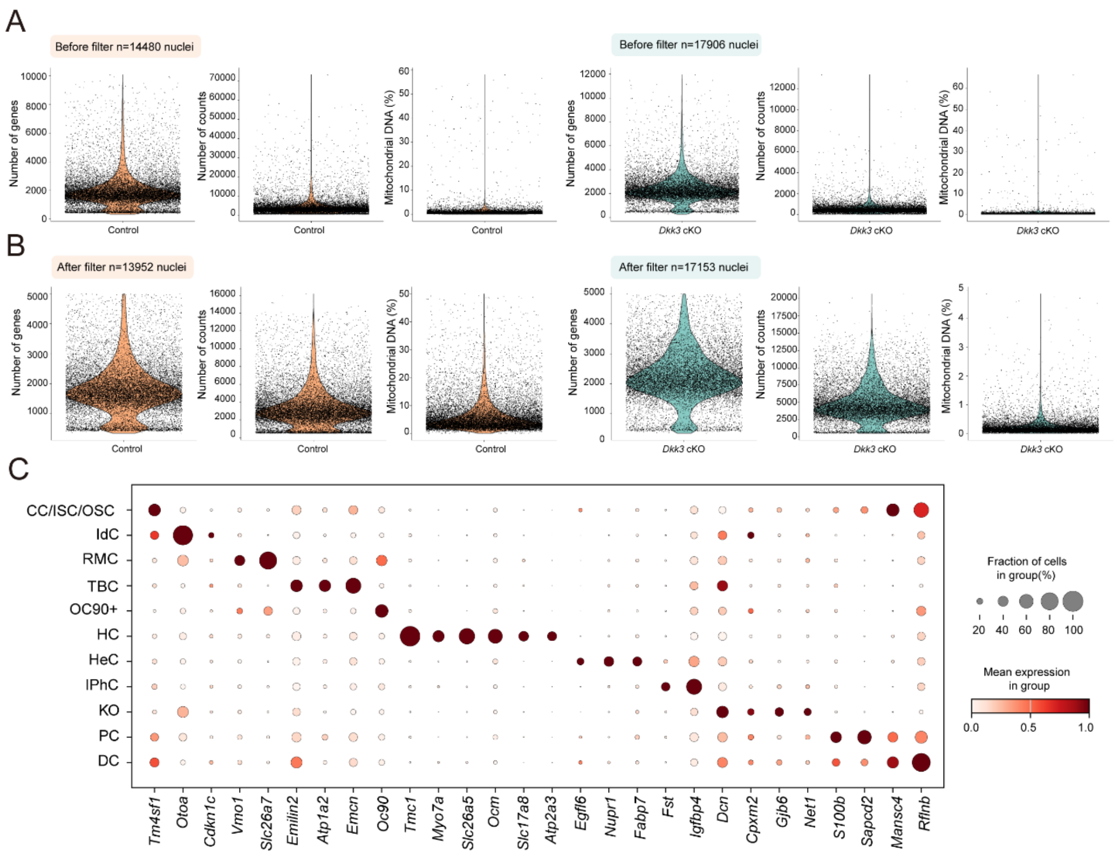

Figure S5. Quality control metrics of snRNA-seq data from control and *Dkk3* cKO groups.

(A–B) Violin plots showing the distribution of counts, genes, and mitochondrial (mt) DNA content per nucleus. (A) Before quality filtering: 14,480 nuclei in the control group and 17,906 nuclei in the *Dkk3* cKO group. (B) After quality filtering, 13,952 nuclei were retained in the control group and 17,153 nuclei in the *Dkk3* cKO group. **(C)** Canonical marker genes used for annotation of cochlear cell types.

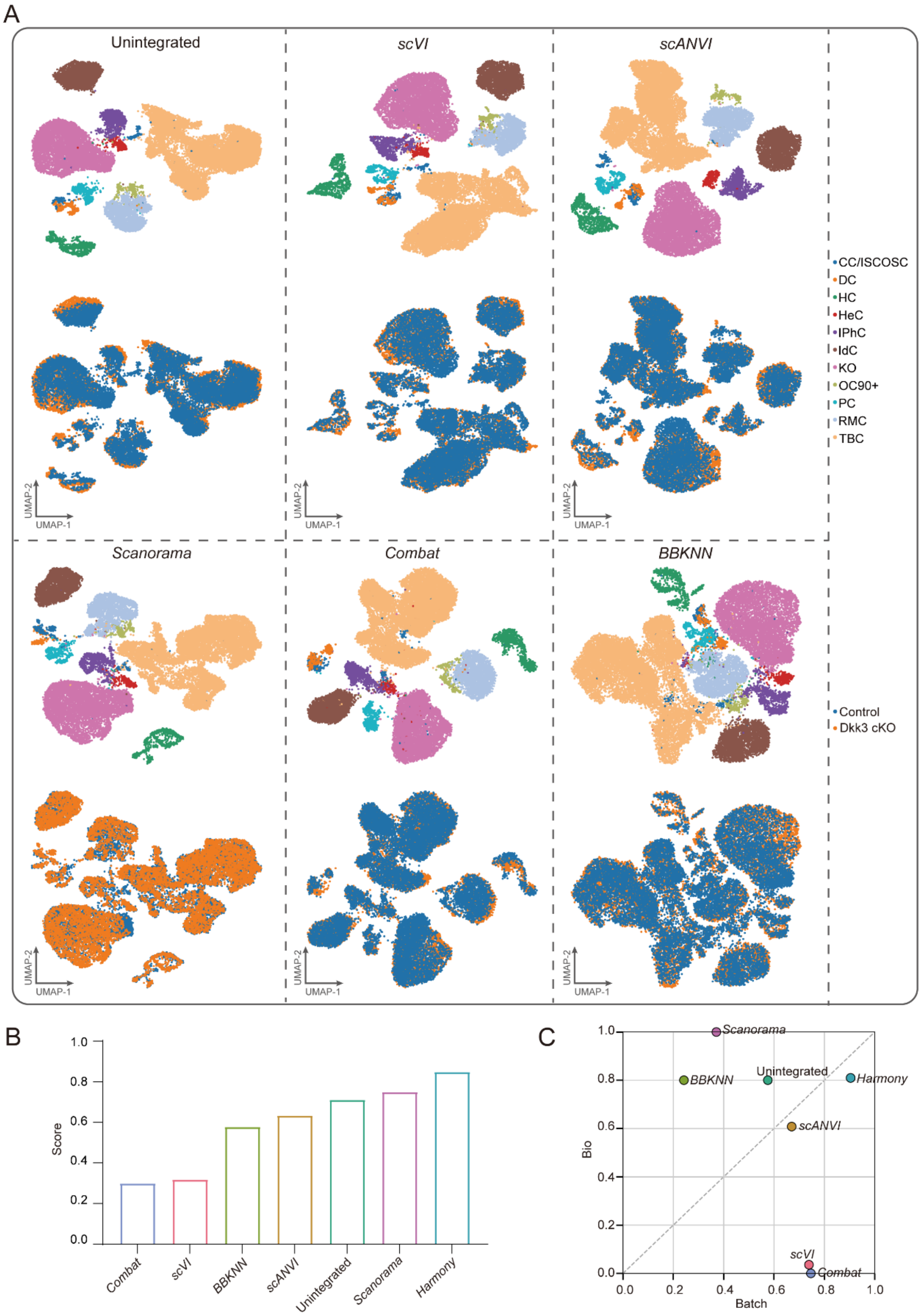

Figure S6. Evaluation of integration algorithms for combining single-cell data from Control and *Dkk3* cKO groups

(A) The UMAP showed that *scVI*, *scANVI*, *Scanorama, ComBat*, and *BBKNN* algorithms integrate the clustering and distribution of cell types in the control and *Dkk3*

cKO groups. **(B-C)** The scores of different algorithms for removing batches between samples while retaining biological significance.

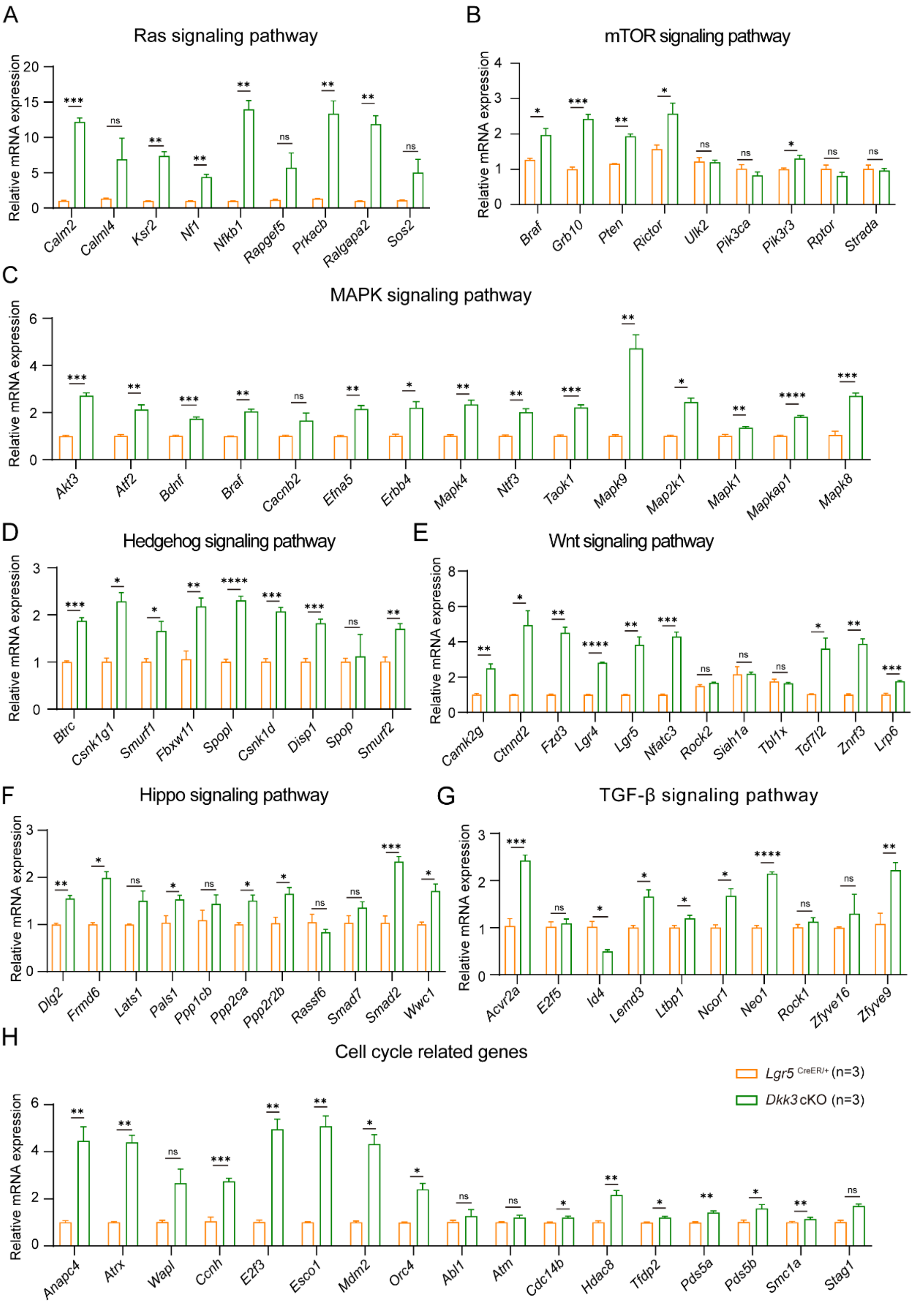

Figure S7. RT-qPCR analysis of signaling pathways in *Dkk3* cKO mouse cochleae. (A-H) RT-qPCR quantification of the expression of Ras signaling pathway genes (A),

mTOR signaling pathway genes (B), MAPK signaling pathway genes (C), Hedgehog signaling pathway genes (D), Wnt signaling pathway genes (E), Hippo signaling pathway genes (F), TGF-β signaling pathway genes (G), and cell cycle ralated genes (H) in the BM of P7 *Lgr5*$^{\mathrm{CreER/+}}$ and *Dkk3* cKO mouse cochleae.

Supporting tables

Supplementary Table 1. 14 DEGs

Abbreviation	Full Name
Capsl	Calcyphosine like
Cntn1	Contactin 1
Col9a1	Collagen type IX alpha 1
Cyr61	Cysteine rich angiogenic inducer 61
Dkk3	Dickkopf WNT signaling pathway inhibitor 3
Fos	FBJ osteosarcoma oncogene
Fosb	FosB proto-oncogene
Mme	Membrane metallo endopeptidase
Ndrg2	NDRG family member 2
Net1	Neuroepithelial cell transforming 1
Otoa	Otoancorin
Serpine2	Serpin family E member 2
Slc6a11	Solute carrier family 6-member 11
Ugdh	UDP-glucose dehydrogenase

Supplementary Table 2. Genotyping primer sequence

Genotyping primers	Sequence
Lgr5 Forward	5'-CTGCTCTCTGCTCCCAGTCT-3'
Lgr5 WT Reverse	5'-ATACCCCATCCCTTTTGA GC-3'
Lgr5 Mut Reverse	5'-GAACTTCAGGGTCAGCTTGC-3'
tdTomato WT Forward	5'-AAGGGAGCTGCAGTGGAGTA-3'
tdTomato WT Reverse	5'-CCGAAAATCTGTGGGAAGTC-3'
tdTomato Mut Forward	5'-GGCATTAAAGCAGCGTATCC-3'
tdTomato Mut Reverse	5'-CTGTTCCTGTACGGCATGG-3'
Dkk3 Forward	5'-GGTCTGACTCACAGATGGACAAA-3'
Dkk3 Reverse	5'-CTCTGCTGCCTAGGAAGATACCA-3'

Supplementary Table 3. The antibodies for Western blot and immunofluorescence

Antibody	Source	Cat#	Application
Anti-Dkk3	Proteintech	10365-1-AP	WB
Anti-beta Actin antibody	Abcam	ab119716	WB
Anti-Myosin7a	Proteus Bioscience	25-6790	IF
Anti-Sox2	R&D systems	AF2018	IF
Anti-CtBP2	BD Biosciences	BD612044	IF
Anti-Prestin	Abcam	ab242128	IF
Anti-Otoferlin	Abcam	ab53233	IF
Anti-Calb1	Abeomics	34-1020	IF
Alexa Fluor™ 488 Phalloidin	Invitrogen	A12379	IF
DAPI	Solarbio	C0060	IF
Alexa Fluor 488	Invitrogen	A-11008	IF
Alexa Fluor 555	Invitrogen	A-21428	IF
Alexa Fluor 568	Invitrogen	A-21124	IF
Alexa Fluor 647	Invitrogen	A-21447	IF

IF: immunofluorescence; WB: Western blot

Supplementary Table S4. Sequences of siRNA for *Dkk3*.

siRNA	Sequence
NC sense	5′ -UUCUCCGAACGUGUCACGUTT- 3′
NC antisense	5′ -ACGUGACACGUUCGGAGAATT- 3′
Dkk3-281 sense	5′ -GGAAGACACUCAGCACAAATT- 3′
Dkk3-281 antisense	5′ -UUUGUGCUGAGUGUCUUCCTT- 3′
Dkk3-419 sense	5′ -GGGAAAUAACACAGUCCAUTT- 3′
Dkk3-419 antisense	5′ -AUGGACUGUGUUAUUUCCCTT- 3′
Dkk3-937 sense	5′ -GCCACAGUCUGGUGUACAUTT- 3′
Dkk3-937 antisense	5′ -AUGUACACCAGACUGUGGCTT- 3′

Supplementary Table S5. Sequences of RT-qPCR primers.

RT-qPCR primers	Sequence
Gapdh Forward	5’-AGGTCGGTGTGAACGGATTTG-3’
Gapdh Reverse	5’- TGTAGACCATGTAGTTGAGGTCA-3’
β-actin Forward	5’-ACGGCCAGGTCATCACTATTG-3’
β-actin Reverse	5’-AGGGGCCGGACTCATCGTA-3’
Dkk3 Forward	5’-CTCGGGGGTATTTTGCTGTGT-3’
Dkk3 Reverse	5’-TCCTCCTGAGGGTAGTTGAGA-3’
Calm2 Forward	5′-GATTAGAGAAGCATTCCGTGTGTT-3′
Calm2 Reverse	5′-CCGTCAATGTCTGCTTCCCTGA-3′
Calml4 Forward	5′-GATCTTCTGGTGTCCATGAGGTG-3′
Calml4 Reverse	5′-GATGGTCAGGAAGGTGGAGAAG-3′
Ksr2 Forward	5′-GTACTGGATGTCTCAGACGTGC-3′
Ksr2 Reverse	5′-CTCGGTGGATAATCAGGAGGTG-3′
Nf1 Forward	5′-CGGCTGCTTTGGAACAATCAGG-3′
Nf1 Reverse	5′-GTGTATCTGCCACAGGCTTGTG-3′
Nfkb1 Forward	5′-GCTGCCAAAGAAGGACACGACA-3′
Nfkb1 Reverse	5′-GGCAGGCTATTGCTCATCACAG-3′
Rapgef5 Forward	5′-AACTGGCTCCAGCACAGAGGAA-3′
Rapgef5 Reverse	5′-TCAGGATCTCCTGGGCTGTACT-3′
Prkacb Forward	5′-CTGAAGAACGGCGTGAGTGACA-3′
Prkacb Reverse	5′-CGAAGTTGCTGGTATCGCCAGA-3′
Ralgapa2 Forward	5′-CACAGATGCTGTGGCTGACTCT-3′
Ralgapa2 Reverse	5′-CCAACCAGTGAGGTTTCCTCCA-3′
Sos2 Forward	5′-CACCAGTGGAATGGCACATCAG-3′
Sos2 Reverse	5′-CTTTGGTCCAGACACTCCCTAC-3′
Braf Forward	5′-CGCCAAGTCAATCATCCACAGAG-3′
Braf Reverse	5′-CACCGAGATTTCACTGTGGCTAG-3′
Grb10 Forward	5′-GGTGAAAGAGGTAGGACGCAAG-3′
Grb10 Reverse	5′-GATGCTGCTTTCTTCCAGGTCAG-3′
Pten Forward	5′-TGAGTTCCCTCAGCCATTGCCT-3′
Pten Reverse	5′-GAGGTTTCCTCTGGTCCTGGTA-3′
Rictor Forward	5′-CAGTGTGAGGTCCTTTCCATCC-3′
Rictor Reverse	5′-GCCATAGATGCTTGCGACTGTG-3′
Ulk2 Forward	5′-GAGGACGAAGACTCTCTACTGG-3′

Ulk2 Reverse	5′-GAGTGCCTACTCCTGGCTTCAT-3′
Pik3ca Forward	5′-CACCTGAACAGACAAGTAGAGGC-3′
Pik3ca Reverse	5′-GCAAAGCATCCATGAAGTCTGGC-3′
Pik3r3 Forward	5′-ACCACGAGTCTCTCGCTCAGTA-3′
Pik3r3 Reverse	5′-CCTGATACTGAGAGTGGAACTCC-3′
Rptor Forward	5′-CTTCCTATCCGTCTTGGCAGAC-3′
Rptor Reverse	5′-CTCCAGACAGATGGCAATCAGG-3′
Strada Forward	5′-ACCGAACCTTCTCGCCTCACTT-3′
Strada Reverse	5′-AGAAGGAGTGGTTCAGCAGGGT-3′
Akt3 Forward	5′-GAGATGGATGCGTCTACAACCC-3′
Akt3 Reverse	5′-TCCACTTGCCTTCTCTCGAACC-3′
Atf2 Forward	5′-CTTCCTCTCCTCAACCAGTCCA-3′
Atf2 Reverse	5′-GAGTCCTAACCAATCCACTGCC-3′
Bdnf Forward	5′-GGCTGACACTTTTGAGCACGTC-3′
Bdnf Reverse	5′-CTCCAAAGGCACTTGACTGCTG-3′
Cacnb2 Forward	5′-CAACTCAGTAAGACCTCTTTGGC-3′
Cacnb2 Reverse	5′-CTGCTACCATCTGGACATTGAGG-3′
Efna5 Forward	5′-TACGCCGTCTACTGGAACAGCA-3′
Efna5 Reverse	5′-GTCTTCTGGGACAGAGTCCTCA-3′
Erbb4 Forward	5′-CAAAGCCAACGTGGAGTTCATGG-3′
Erbb4 Reverse	5′-CTGCGTAACCAACTGGATAGTGG-3′
Mapk4 Forward	5′-CGCCAACATCTTCATCAGCACG-3′
Mapk4 Reverse	5′-CCACTTTGTCACCAACCCTTCTG-3′
Mapk8 Forward	5′-CGCCTTATGTGGTGACTCGCTA-3′
Mapk8 Reverse	5′-TCCTGGAAAGAGGATTTTGTGGC-3′
Ntf3 Forward	5′-CTACTACGGCAACAGAGACGCT-3′
Ntf3 Reverse	5′-GGTGAGGTTCTATTGGCTACCAC-3′
Taok1 Forward	5′-GGCGACATAACTTGGAACAGGAC-3′
Taok1 Reverse	5′-GTGTTGAGATGGCGAAACTCCAG-3′
Mapk9 Forward	5′-GTCAGTGGGTTGCATCATGGGA-3′
Mapk9 Reverse	5′-ACTCTGCGGATGGTGTTCCTAG-3′
Map2k1 Forward	5′-AAGGTCTCCCACAAGCCATCTG-3′
Map2k1 Reverse	5′-AGTTGCACTCGTGCAGTACCTG-3′
Mapk1 Forward	5′-TCAAGCCTTCCAACCTCCTGCT-3′
Mapk1 Reverse	5′-AGCTCTGTACCAACGTGTGGCT-3′

Mapkap1 Forward	5′-CTGGAGAAGCAGAGTGAGCCTA-3′
Mapkap1 Reverse	5′-CGAAAACTCCGTCTGCCCTTGA-3′
Mapk8 Forward	5′-CGCCTTATGTGGTGACTCGCTA-3′
Mapk8 Reverse	5′-TCCTGGAAAGAGGATTTTGTGGC-3′
Btrc Forward	5′-GCAGTACGATGAGAGGGTGATC-3′
Btrc Reverse	5′-CAGAACGGCTTCACAGTGGTGA-3′
Csnk1g1 Forward	5′-TACTTCCTCCGAGGCAGCCTAC-3′
Csnk1g1 Reverse	5′-TCGAAGGTAGGTTGCCATCTCC-3′
Smurf1 Forward	5′-GGAGGAAGGTTTGGACTATGGTG-3′
Smurf1 Reverse	5′-CCGTGGAATACTGGAAGAGTCC-3′
Fbxw11 Forward	5′-TGCCTCCAGTATGATGAGCGAG-3′
Fbxw11 Reverse	5′-GTCCATTGCTGAAGCGTAAGTGC-3′
Spopl Forward	5′-GGTCTCCTTCCAGATGACAAGC-3′
Spopl Reverse	5′-CTGCTAGTCGACATTCTGGCAC-3′
Csnk1d Forward	5′-CCATCAACACGCACCTTGGCAT-3′
Csnk1d Reverse	5′-CATACTTCTGCCTCTTGGTGGC-3′
Disp1 Forward	5′-GGTCAGACGATCACCATGAGAG-3′
Disp1 Reverse	5′-CCTCCTGCTGAAGCAAACACTAC-3′
Spop Forward	5′-GCTGTCCAAAGAGTGAAGTTCGG-3′
Spop Reverse	5′-GCACGAACCTATAAGCTCGCTG-3′
Smurf2 Forward	5′-CCAATGCCATCAACCGCCTCAA-3′
Smurf2 Reverse	5′-GTGCCTATTCGGTCTCTGGACT-3′
Camk2g Forward	5′-GGACACAGTCACTCCTGAAGCT-3′
Camk2g Reverse	5′-TCTACCGTCTCTTGGCGATGCA-3′
Ctnnd2 Forward	5′-GTGAGACTCCTTCGCAAGACCA-3′
Ctnnd2 Reverse	5′-GGATAATCACCGCATTGGTCAGC-3′
Fzd3 Forward	5′-GCAAAGTGAGCAGCTACCATGG-3′
Fzd3 Reverse	5′-AGCCGATGAGAACTACTGTGCC-3′
Lgr4 Forward	5′-CAAATGCCACCAGCACTGCTGA-3′
Lgr4 Reverse	5′-CCACACTGTAAGGCGAATCATCC-3′
Lgr5 Forward	5′-AGAGCCTGATACCATCTGCAAAC-3′
Lgr5 Reverse	5′-TGAAGGTCGTCCACACTGTTGC-3′
Nfatc3 Forward	5′-CAGTTGCTCTGTCAATGGAGGC-3′
Nfatc3 Reverse	5′-TTCCTTCAGCCTCCCAATGAGG-3′
Rock2 Forward	5′-GTGACCTCAAACAGTCTCAGCAG-3′

Rock2 Reverse	5′-GACAACGCTTCTGAGTTTCCTGC-3′
Siah1a Forward	5′-ACTCTGCCACACACCGAAAAGG-3′
Siah1a Reverse	5′-ATGACGGCATCCAAGGAGCCTT-3′
Tbl1x Forward	5′-CCGAGTAAAGCCACAGTCCTTC-3′
Tbl1x Reverse	5′-TATCCTCGCAGTGGAGTCTCCA-3′
Tcf7l2 Forward	5′-CGCTGACAGTCAACGCATCTATG-3′
Tcf7l2 Reverse	5′-GGAGGATTCCTGCTTGACTGTC-3′
Znrf3 Forward	5′-CTCATCGGTTCCACAGGAAGTG-3′
Znrf3 Reverse	5′-CGTGTAAGGTTGCTTGTCTCCAC-3′
Lrp6 Forward	5′-CTGAATGCTGACAACAGGACCTG-3′
Lrp6 Reverse	5′-GACGTTCCGAAGGCTGTGGATA-3′
Dlg2 Forward	5′-TGCTTCTCCCAGGCACTATTCC-3′
Dlg2 Reverse	5′-CGTTGGAGAGTCACTGAAGGCT-3′
Frmd6 Forward	5′-GTTGAGAGTGGAGGCAAAGACC-3′
Frmd6 Reverse	5′-CACATCTCTGGGCTGACTTCCA-3′
Lats1 Forward	5′-TGGGACAACCTCCTTTCTTGGC-3′
Lats1 Reverse	5′-TGAGGTCAGAGGCTTCAGGACT-3′
Pals1 Forward	5′-GCGTTGTTAGCCAAGGAAGGCA-3′
Pals1 Reverse	5′-CGAAGTAGTGTCCGTTGTTCTGC-3′
Ppp1cb Forward	5′-CTCGTGAAATCTTTCTCAGCCAG-3′
Ppp1cb Reverse	5′-TGGCTTCTGGTGGAAAACCTCC-3′
Ppp2ca Forward	5′-TCTTCCTCTCACTGCCTTGGTG-3′
Ppp2ca Reverse	5′-GAGGAACTTCCTGTAGGCGATC-3′
Ppp2r2b Forward	5′-AGTCAGTGAGCGGGATAAGAGG-3′
Ppp2r2b Reverse	5′-GCATTGGCAAACACTCTTCGTGG-3′
Rassf6 Forward	5′-GACCTTTACCGTATCAGCGAGC-3′
Rassf6 Reverse	5′-GCTCTTCGTCTGCGTATGGTGT-3′
Smad7 Forward	5′-GTCCAGATGCTGTACCTTCCTC-3′
Smad7 Reverse	5′-GCGAGTCTTCTCCTCCCAGTAT-3′
Smad2 Forward	5′-CCAACTGTAACCAGAGATACGGC-3′
Smad2 Reverse	5′-AACCCTGGTTGACAGACTGAGC-3′
Wwc1 Forward	5′-AGCCATCACCTGCGGAGAAAAG-3′
Wwc1 Reverse	5′-TGGACCATAGGTCGGAGTGTGA-3′
Acvr2a Forward	5′-AAGTTCGAGGCTGGCAAGTCTG-3′
Acvr2a Reverse	5′-CCTCAGAAATGCGTCCCTTTGG-3′

E2f5 Forward	5′-AGAAGTTGTGGCTACAGCAAAGC-3′
E2f5 Reverse	5′-AGCTGTGTACCAGAAGGTGCCT-3′
Id4 Forward	5′-AGTGCGATATGAACGACTGCTAC-3′
Id4 Reverse	5′-AGCAAAGCAGGGTGAGTCTCCA-3′
Lemd3 Forward	5′-AGATGCCCTTCTGGTGCCGTTT-3′
Lemd3 Reverse	5′-CCTCCTTAGTCCAGCGGTATCT-3′
Ltbp1 Forward	5′-TGCCTGTGGAAGTAGCTCCTGA-3′
Ltbp1 Reverse	5′-AGTGTCCTGCTCCGCAAATGTC-3′
Ncor1 Forward	5′-GCCAGCAAAATCTCAGTGGAGAC-3′
Ncor1 Reverse	5′-CCTCTGTTGGTATTCCAGCCTG-3′
Neo1 Forward	5′-TGACATGGCGTACACCTGCATC-3′
Neo1 Reverse	5′-GGCTGGTATTCTCAACACGCTC-3′
Rock1 Forward	5′-CACGCCTAACTGACAAGCACCA-3′
Rock1 Reverse	5′-CAGGTCAACATCTAGCATGGAAC-3′
Zfyve16 Forward	5′-CAGCCACTTCAGGAGACCCAAA-3′
Zfyve16 Reverse	5′-CTTCACCATTCGGCAGTATGCC-3′
Zfyve9 Forward	5′-TGTAGCCACCAGTCCTCTTCCA-3′
Zfyve9 Reverse	5′-ACACCAGTGGAGATGAGGATGG-3′
Anapc4 Forward	5′-GGCTGCTAGAAAGCATGAGAGC-3′
Anapc4 Reverse	5′-CTCCTCATCGTCTGAAGACTCG-3′
Atrx Forward	5′-GTGAATCTGAGGATGAACAGCGG-3′
Atrx Reverse	5′-CCTCTTGAACCTTAATGCGCCG-3′
Wapl Forward	5′-TGCCTGAGAGTGTGAAGAAGCC-3′
Wapl Reverse	5′-GTCTGGATGGTTCCAATGCCTG-3′
Ccnh Forward	5′-ACTTGCCTGTCACAGTTACTGGA-3′
Ccnh Reverse	5′-GAATGACACCGCTCCAGCTTCT-3′
E2f3 Forward	5′-GTCCAGAAGAGACGGAAACACAC-3′
E2f3 Reverse	5′-GCTGTAGAAACCGAGCAGTCAC-3′
Esco1 Forward	5′-GCACCTGCTTTTCCACAACCAG-3′
Esco1 Reverse	5′-CAGGGCATACTTTGGGTCTTCAG-3′
Mdm2 Forward	5′-CCGAGTTTCTCTGTGAAGGAGC-3′
Mdm2 Reverse	5′-GTCTGCTCTCACTCAGCGATGT-3′
Orc4 Forward	5′-AGTCCACATAGCAACCTCTTTGG-3′
Orc4 Reverse	5′-GTGGTCTTTCCTGATCCTCGTG-3′
Abl1 Forward	5′-TGAGCAGAAAGATGCGCCTGAC-3′

Abl1 Reverse	5′-CGCTCATCTTCATTTAGGCTGCC-3′
Atm Forward	5′-CCAAGATGGCAGTGAACCAGAC-3′
Atm Reverse	5′-ATGCTGGACAGCTATGGTGGAG-3′
Cdc14b Forward	5′-GACACAAGGAGACAGACTTCGG-3′
Cdc14b Reverse	5′-CACAATCCACCAGATGGCAACAC-3′
Hdac8 Forward	5′-GTCAGCCAAGAAGGTGATGAGG-3′
Hdac8 Reverse	5′-ACACTTCCCGTCAATCAGGCAC-3′
Tfdp2 Forward	5′-GTCATAGACTGCAGCATCTCCAG-3′
Tfdp2 Reverse	5′-GAGCATTTGCCTGACTCCAGAC-3′
Pds5a Forward	5′-GCTCAGGAACCTTGCTACCATG-3′
Pds5a Reverse	5′-GGAGAAGCAACTTCACAAGTGCC-3′
Pds5b Forward	5′-ATGTCACGCCTGAGACTTGCTG-3′
Pds5b Reverse	5′-GTTTCTGAGCGAACACCTGCCT-3′
Smc1a Forward	5′-ACGCATTGCCTTTGGAGGTCAC-3′
Smc1a Reverse	5′-GCCGTGCTTTTGCCTTGAGATC-3′
Stag1 Forward	5′-GAACATGCAGCCTACTTGGTGG-3′
Stag1 Reverse	5′-AGAGCACTCTCTTGACGGTCAG-3′
